# Nanostructure Nickel-Based Selenides as Cathode Materials for Hybrid Battery-Supercapacitors

**DOI:** 10.3389/fchem.2020.611032

**Published:** 2021-02-02

**Authors:** Haocheng Sun, Chensheng Wang, Zhiqiang Qi, Wenliang Hu, Zhijie Zhang

**Affiliations:** Huazhong Institute of Electro-Optics, Wuhan National Laboratory for Optoelectronics, Wuhan, China

**Keywords:** material modification, cathode materials, battery-supercapacitors, nickel-based selenides, carbon substrate

## Abstract

Supercapacitors (SCs) have attracted many attentions and already became part of some high-power derived devices such as Tesla’s electric cars because of their higher power density. Among all types of electrical energy storage devices, battery-supercapacitors are the most promising for superior performance characteristics, including short charging time, high power density, safety, easy fabrication procedures, and long operational life. An SC usually consists of two foremost components, namely electrode materials, and electrolyte. The selection of appropriate electrode materials with rational nanostructured designs have resulted in improved electrochemical properties for high performance and has reduced the cost of SCs. In this review, we mainly spotlight the nickel-based selenides nanostructured which applied as high-performance cathode materials for SCs. Different nickel-based selenides materials are highlighted in various categories, such as nickel-cobalt-based bimetallic chalcogenides and nickel-M based selenides. Also, we mentioned material modification for this material type. Finally, the designing strategy and future improvements on nickel-based selenides materials for the application of SCs are also discussed.

## Introduction

The rapid advance of the global economy results in the over-exploitation and over-consumption of primary energy, indicating an increasingly severe environmental pollution. Consequently, the development of renewable clean energy is extremely urgent, and the development of efficient and safe energy storage devices is very important. The Super Capacitor (SC) has more advantages compared with secondary batteries and traditional capacitors, including high power density, fast charge and discharge process, excellent cycle stability, quick dynamic response, longer life than ordinary batteries, and low cost, environmental friendliness, and safety. Hence, given all the advantages of the SC, it has aroused widespread concern among researchers. The main parts of SC are positive electrode material, negative electrode material, electrolyte, and separator. SC has high power density, but its energy density has a certain gap compared with secondary batteries (such as lithium ion batteries, sodium ion batteries, etc.). Because batteries have high energy density, if the advantages of batteries and the SC are combined -- battery-type electrode materials and capacitive electrode materials are asymmetrically packaged into hybrid SC -- the inherent advantages of different types of materials are given full play, which could further improve the capacitance and energy density of the hybrid device.

**FIGURE 1:**
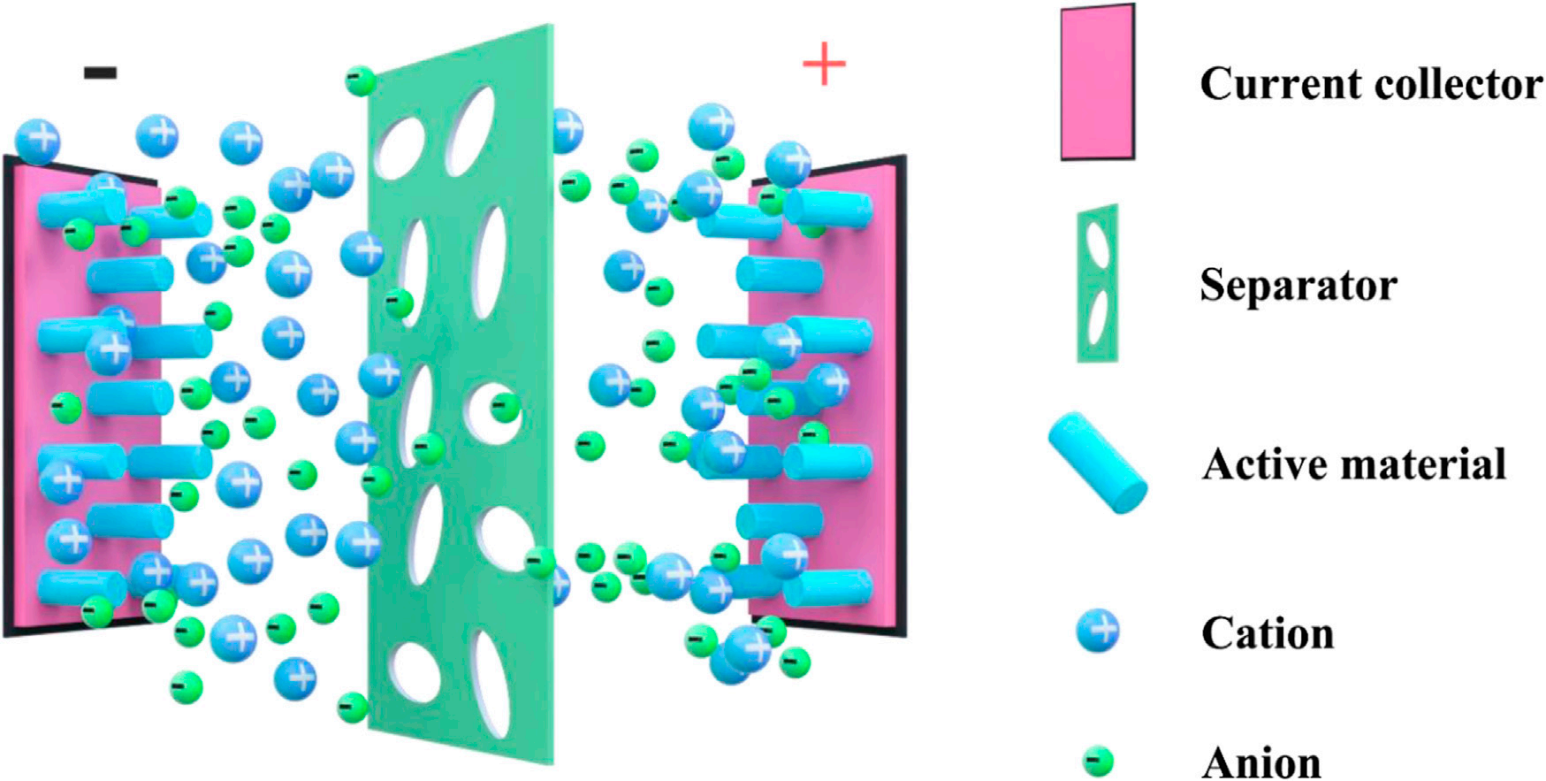
Basic structure of hybrid battery-supercapacitors.

Hybrid SC, also known as Battery-SC Hybrid Devices (BSHs) (Winter and Brodd, 2004), (Zuo et al., 2017) use capacitive electrode materials as the “power source” of the device, and regard battery-type electrode materials as the “energy source” of the device (Figure 1). These two materials’ synergistic collaboration enables the rapid charge transfer capability of the electric double layer material and the superior capacitance capability of the battery-type material (SIMON and GOGOTSI, 2008). As a Faraday pseudocapacitor or battery electrode material, metal compounds have excellent electrochemical properties. As early as 1971, S. Trasatti and Buzzanc conducted research on the electrochemical properties of RuO_2_ and found that RuO_2_ has the advantages of high energy density and power density. Nonetheless, it is a precious metal oxide with disadvantages such as toxicity, which restricts its commercial use. In recent years, studies have demonstrated that nickel-based compounds are of excellent properties (such as nickel oxide, nickel hydroxide, nickel selenide, etc.), which could be a substitute for RuO_2_ and is worth in-depth research.

Supercapacitors store electric energy through redox reaction between electrolyte and active material. They are much larger than double-layer capacitance of carbon materials, so they have become a hot research topic. Due to the high cost of precious metals and the scarcity of resources, transition metal oxides with good cost-effectiveness have become the focus of research, such as manganese dioxide, iron oxide, cobalt oxide, nickel oxide, tin oxide, etc. In addition to low cost, these materials have no pollution to the environment and exhibit excellent specific capacity in neutral and alkaline electrolytes. However, their cycling stability is not as good as that of noble metal oxides and carbon materials. Therefore, transition metal oxides are gradually compounded with carbon materials or different transition metal oxides. The research results show that these methods can effectively improve the electrochemical performance of metal oxides.

Kolathodi et al. (2015) chose the solvent gel method to prepare NiO nanofibers. Then the nanofibers were used as battery-type electrodes and were assembled with activated carbon electrodes to prepare hybrid SCs, which excel at performance and are of high energy density as high as 43.75 wh/kg. Ge et al., (2012) prepared graphene/NiO composite electrodes with a 3D structure by hydrothermal method and the material obtained have a capacitance value of 346 F/g. Lu et al. (2011) deposited ultra-thin Ni(OH)_2_ nanosheets on foam nickel by hydrothermal method. The composite material showed a capacity of 2675 F/g. Besides, after 500 cycles at 30 mA/cm^2^, the attenuation rate was less than 4%. Yang et al. (2008) used electrodeposition to prepare Ni(OH)_2_ with H-dimensional porous structure on foamed nickel. Its capacity was as high as 3152 F/g at 4 A/g. With the chemical bath deposition method, Yan et al. (2013) synthesized a porous nickel oxide hollow sphere with a thickness of 10 nm, whose capacity was still 346 F/g after 2,000 cycles at 1 A/g. All the above reports illustrate that nickel oxide/nickel hydroxide, as an electrode material for SCs, has a high theoretical specific capacitance and excellent capacitance characteristics. However, as a p-type semiconductor electrode material, nickel oxide/nickel hydroxide also has the disadvantage of poor electrical conductivity. For instance, the polarization phenomenon of the material is serious, the rate performance is poor, and the cycle life is short under the conditions of high current density charge and discharge.

**FIGURE 2:**
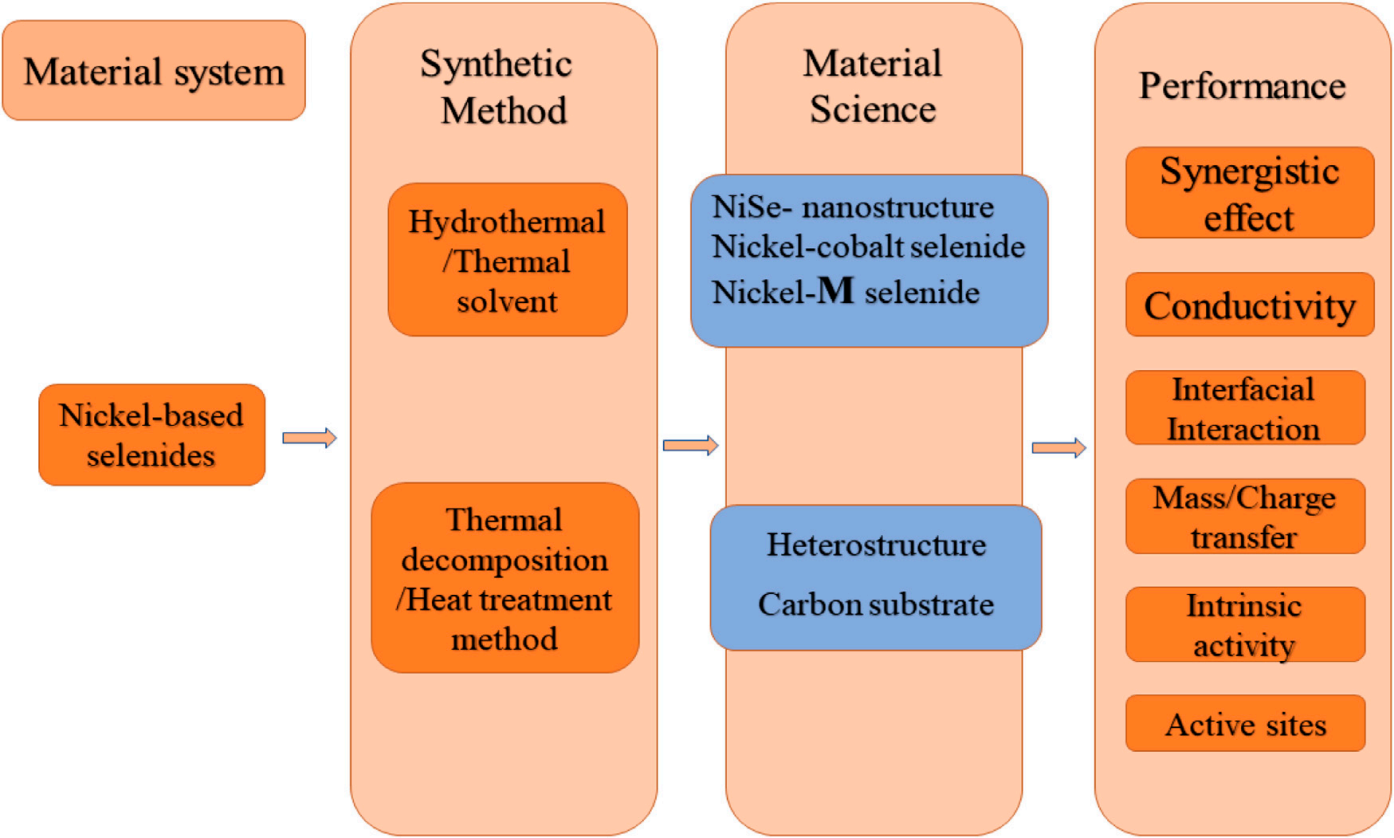
An illustration of the key performance metrics, the affecting factors in evaluation of SCs, where nickel-based selenides are shown to play major roles in development of high performance supercapacitors.

Since selenium, sulfur, and oxygen belong to the same main group element, selenide has chemical properties like sulfides and oxides and has stronger metallic properties. Similar to oxides, transition metal selenides have attracted increasing attention because of their high theoretical specific capacity, low cost, rich crust content, and easy manufacturing (Figure 2). For example, Wang (Tian et al., 2017) used a one-pot hydrothermal method to synthesize NiSe nanorod arrays on nickel foam, which has an ultra-high specific capacitance of 6.81 F/cm^2^ and excellent cycle stability when the current density is 5 mA/cm^2^. Meng et al. (2019) designed a reasonable single-step hydrothermal method to obtain pyramid-shaped nanostructured NiSe_2_. Kirubasankar et al. (2018) obtained NiSe nanoparticle materials with excellent consistency on graphene nanosheets by *in-situ* hydrothermal method Consequently, nickel-based selenide could be regarded as promising electrode material in SC.

The synthesis of nickel-based selenide nanostructures is a comprehensive field that combines traditional physical and chemical methods and a variety of emerging methods. Although the synthesis steps cover a wide range, in general, the main control directions can be classified into the following two categories.

(1) Control the size, composition and morphology of nanoclusters, including aerosols, powders, semiconductor quantum dots and other nano-components.
(2) Control the interface and distribution of each nano-component in the composite material. The above two aspects are inseparable. However, it is very important to understand how to control the nucleation and growth processes of the nanostructure elements during the entire synthesis and assembly process.

**FIGURE 3:**
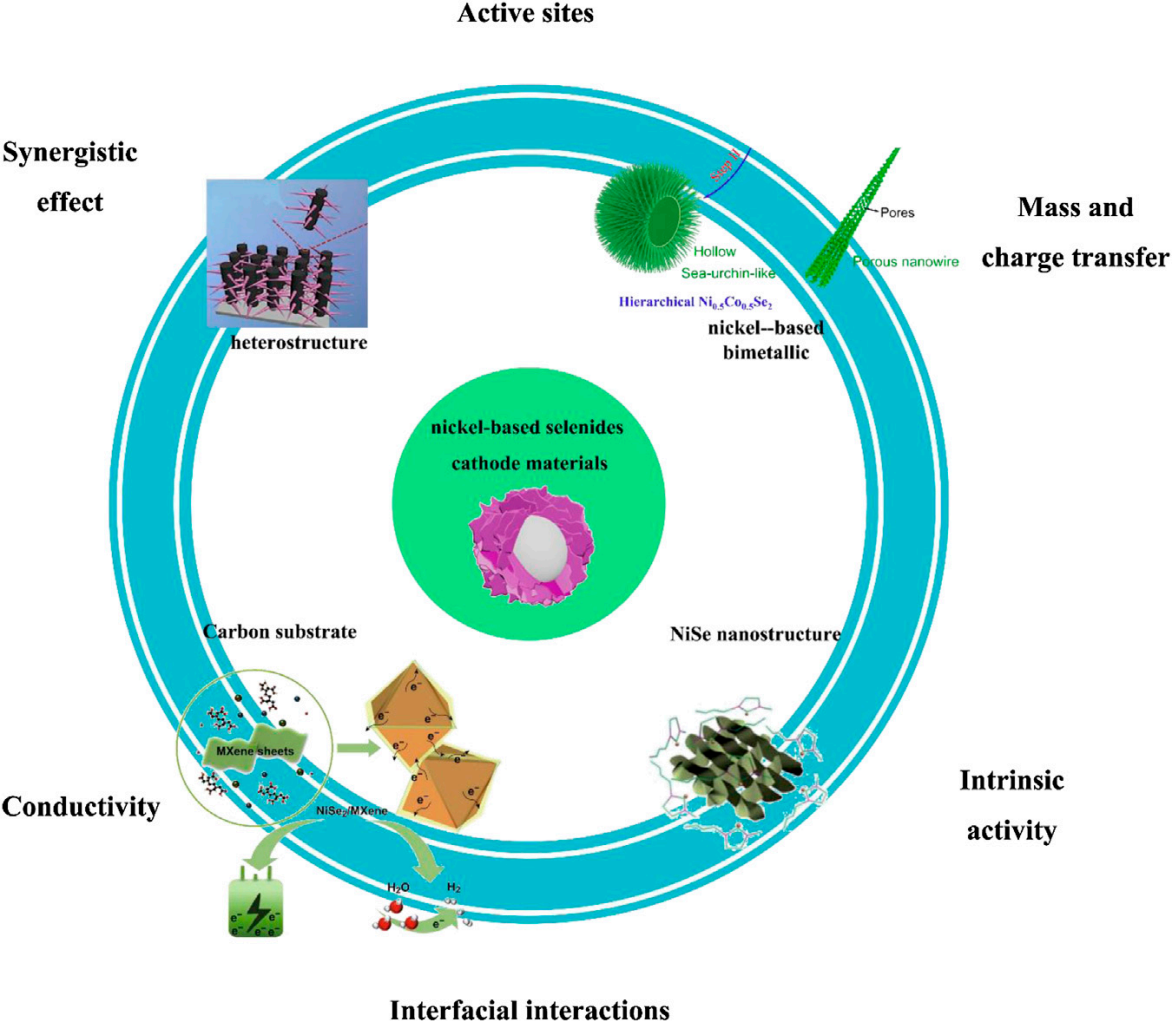
nickel-based selenides as cathode materials for SC.

There are problems, such as difficult synthesis methods and complex preparation steps, in the process of exploring nickel selenide as an SC electrode material. In recent years, after continuous exploration by scientific researchers, significant progress has been made in the research of nickel selenide as an electrode material for SCs (Figure 3). Tang et al., (2015) adopted a one-step hydrothermal method to grow NiSe nanowires as electrode materials on the surface of a foamed nickel substrate. The results indicated that at a current density of 5 A/g, the maximum specific capacitance of the NiSe electrode could reach 1790 F/g, and the area-specific capacitance was 5.01 F/cm^2^. Arul (Arul and Han, 2016) used a hydrothermal method to prepare a Nize_2_ electrode material with a two-dimensional hexagonal structure. When the current density is 1 mA/cm^2^, the capacitance value is 75 F/g. And after 5,000 cycles, the capacitance attenuates only 6% compared with the initial value, which implies that the NiSe_2_ electrode material is a kind of electrode material with high capacity and stable cycle. Wang et al. (2017) prepared a short cubic structure of NiSe_2_ single crystal by hydrothermal method, and conducted an in-depth exploration of its electrochemical performance. With the continuous deepening of research, the direction has turned to explore the simple preparation method and excellent electrochemical performance of nickel selenide as an SC electrode material, which sheds some light on the development of high-performance hybrid SCs.

Due to many nickel-based selenides structures and types, optimization methods for increasing the energy density of the SC have received extensive attention in recent years. However, there is currently no complete summary of nickel-based selenides. Consequently, this paper will summarize the preparation methods of nickel-based selenide, nickel-based selenide cathode materials (nickel selenide, nickel-based bimetallic selenide), and the performance optimization path of nickel-based selenide.

## Methods

At present, there are two main methods for preparing nickel-based SC electrodes: hydrothermal method/thermal solvent method and thermal decomposition method/heat treatment method (two-step method).

### Hydrothermal Method

**FIGURE 4:**
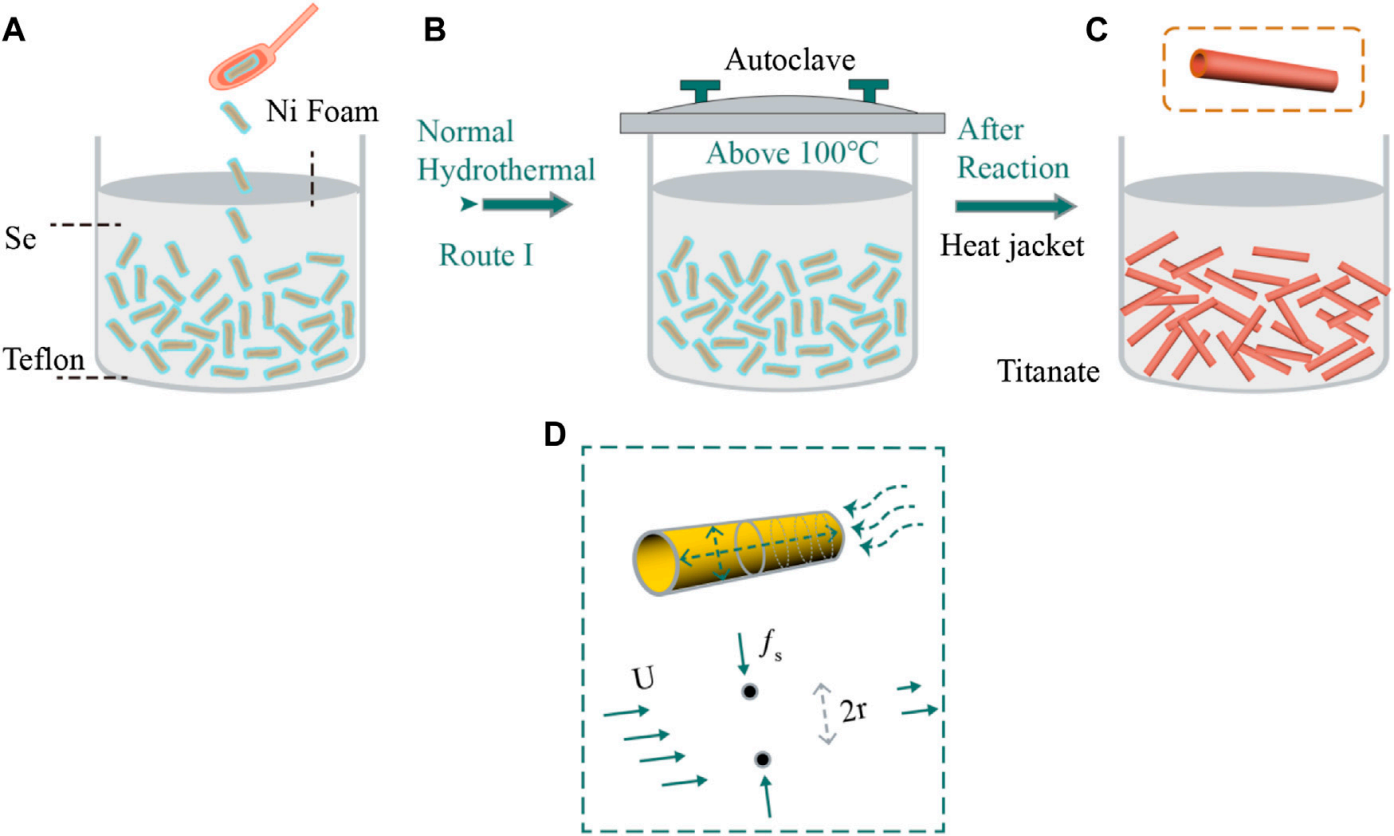
schematic diagram of typical hydrothermal method mechanism.

Since 1960, the hydrothermal method has been widely used to synthesize various nanomaterials with specific size and morphology. The specific method is as follows (Figure 4). Then, in the hydrothermal reaction system, water, as a reaction medium, dissolves inorganic salts, and is sealed in a reactor with a polytetrafluoroethylene substrate to participate in the reaction. The reaction temperature is usually higher than 100°C, which is conducive to the autogenous pressure generated by the closed system to obtain a high-pressure state. The increase of reaction temperature causes corresponding changes in the properties of water: 1) the vapor pressure increases; 2) the surface tension decreases; 3) the density falls; 4) the viscosity declines; 5) the ion product increases (Gao et al., 2013). Moreover, the dissociation constant of water increases with the increase in temperature, which intensifies the hydrothermal reaction rate.

Currently, many nickel-based selenides with different sizes and morphologies have been prepared with this method. For example, Du et al. (2017) have grown a honeycomb-like ultra-thin NiSe nanosheet structure on the surface of the foamed nickel substrate by a typical one-step hydrothermal method as an electrode material. The sheet structure serves as the electrode material. Karthikeyan et al. (2014) mixed Ni(NO_3_)_2_·6H_2_O, CH_4_NS, and EDTA in different proportions to prepare hierarchical spherical NiS, NiS_2_, Ni_17_S_18_, Ni_3_S_4_, and other compounds. During the formation of Nise_2_ compounds, the main reactions are as follows

$${\text{SeO}}_{2}{+\text{HOCH}}_{2}{\text{CH}}_{2}{\text{OCH}}_{2}{\text{CH}}_{2}\text{OH}\to {\text{Se}+\text{OHCCH}}_{2}{\text{OCH}}_{2}{\text{CHO}+2\text{H}}_{2}\text{O}$$ (1)

$$3\text{Se}+6{\text{OH}}^{-}\to 2{\text{Se}}^{2-}{+\text{SeO}}_{3}^{2-}+3{\text{H}}_{2}\text{O}$$ (2)

$${\text{Se}}^{2-}+\text{Se}\to {\text{Se}}_{2}^{2-}$$ (3)

$${\text{Ni}}^{2+}{+\text{Se}}_{2}^{2-}\to {\text{NiSe}}_{2}$$ (4)

Analyzing the experimental phenomena and results, the final morphology of the NiSe_2_ compound is generally dendritic or octahedral under the condition of a longer reaction time or a higher reaction temperature. Therefore, the growth mechanism of NiSe_2_ compound can be described in Figure 4. During the formation of NiSe_2_ compound, SeO_2_ is reduced to produce Se^2-^, Se^2-^ and Se produce Se_2_^2-^, and finally NiSe_2_ is produced. Add NaOH deionized aqueous solution to the solution. When NaOH is fully mixed with NiCl_2_•6H_2_O and SeO_2_, NaOH can accelerate the rate of DEG reduction of SeO_2_, thereby speeding up the synthesis of NiSe_2_ compounds. Through the process, samples with more regular shapes and smaller sizes are generated.

Meng et al. (2019) designed a reasonable single-step hydrothermal method to obtain pyramid-shaped nanostructured NiSe_2_. Further, Kirubasankar et al. (2018) used a single-step hydrothermal method to grow NiSe nanocomposite electrode *in situ* on graphene. Ye et al. (2018) had synthesized a self-supporting Ni_0.85_Se nanosheet array structure on a carbon fiber fabric through a direct and gentle hydrothermal method.

As for the hot solvent method, Gu et al. (2019) used a hydrothermal method to prepare Ni(OH)_2_ precursor and then chose the hot solvent method to carry out splenization reaction to prepare a NiSe_2_ nanoparticle decorated with nitrogen-doped reduced graphene oxide.

According to the above literature, the hydrothermal method occupies a considerable proportion of the preparation and synthesis of nickel-based compound materials. In general, the hydrothermal method/hot solvent method has the following characteristics:

(1) Under hydrothermal and thermal solvent synthesis conditions, the reaction performance and activation performance of the reactants are improved, and products that are difficult to obtain by solid-phase reactions can be synthesized. Besides, a series of new synthesis methods are derived;
(2) The hydrothermal method and the hot solvent method have low reaction temperature, uniform pressure distribution, and liquid flow state, which is beneficial to the growth of crystals with fewer defects, good orientation, pure phase, regular morphology, high crystallinity, and narrow particle size distribution;
(3) Hydrothermal and thermal solvent methods can facilitate the synthesis of intermediate, metastable and special phase products, which is conducive to the application of hydrothermal and thermal solvent methods to the synthesis and utilization of some special metastable structures and condensed products in;
(4) The synthesis conditions of the hydrothermal method and the hot solvent method are easy to adjust, which is very beneficial to the synthesis of low-valence, intermediate valence, and some special valence compounds, and the doping is uniform.

### Thermal Decomposition

Thermal decomposition synthesis method, also known as sintering method, is a popular non-injection synthesis method, which originated from the oxidation treatment method of preparing nickel oxide nanomaterials in early research. Since selenide is easily oxidized in the air, a suitable precursor nickel-based nanostructure is first prepared, and then heated and calcined in a tube furnace under the protection of inert gas or a reducing atmosphere. The position is divided into upstream and downstream. The selenium powder is placed upstream and the reaction precursor is placed downstream. The gasified selenium powder is taken as a carrier to the downstream precursor for reaction to complete the splenization of the electrode material. When a reasonable nanostructure can be prepared, the thermal decomposition method/heat treatment method is extremely effective for preparing metal selenide nanocrystalline materials. The reason is that the selenide nanocrystalline material can be directly decomposed at a certain temperature, and then selenized to form transition metal selenide while retaining some of the structural characteristics of the precursor. Consequently, this means can adjust the size and morphology of metal selenide nanocrystalline materials by changing the ratio of starting reagents (including precursors, ligands, and solvents), reaction temperature, and reaction time. So far, metal selenides (containing bimetallic nickel-based selenides) with various morphologies and sizes have been synthesized by this method.

**FIGURE 5:**
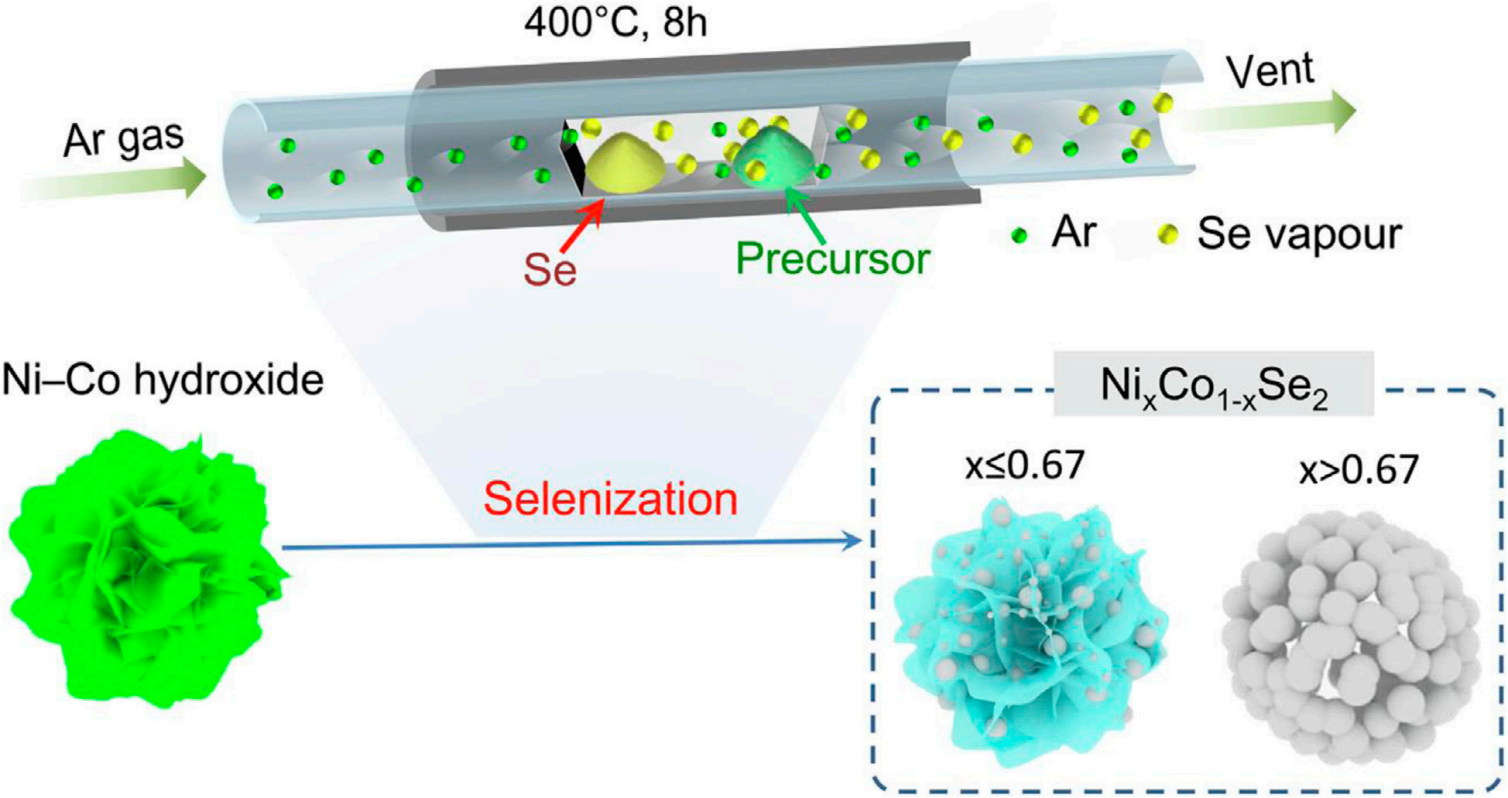
schematic diagram of typical thermal decomposition synthesis method.

**FIGURE 6:**
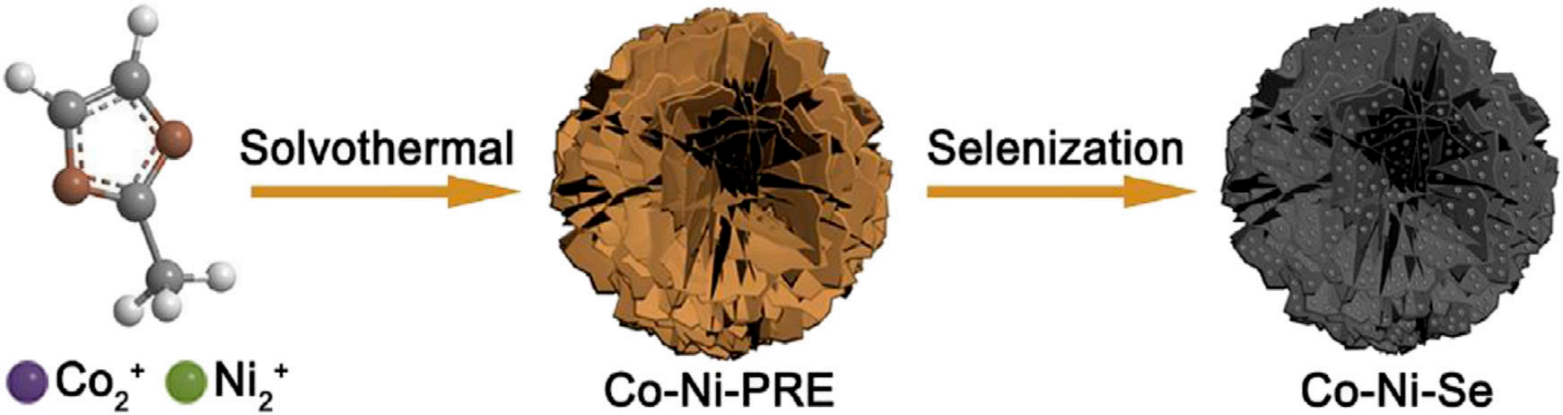
Schematic illustration of Co-Ni-Se NFs formation.

Hu et al. (2020) proposed an environmentally friendly heat treatment splenization method -- a layered nickel-cobalt selenide nanoparticle/nanosheet electrode material was prepared by controlling the sample recrystallization during the splenization process (Figure 5). The electrode material has a specific capacitance of 447 C/g at 1 A/g, and it still retains 97% of the initial value after 2,000 cycles. Huang et al. (2020) first prepared a Ni-Co precursor in the shape of a peony flower by a special bottom-up reaction hot solvent method. Afterward, with the typical thermal decomposition method, the precursor and selenium powder were placed on both ends of the quartz boat, and then the quartz boat was transferred to the tube furnace. The thermal decomposition method was used to carry out splenization at 350°C in a nitrogen atmosphere to obtain a Ni-Co selenide material with spherical petals (Figure 6).

**FIGURE 7:**
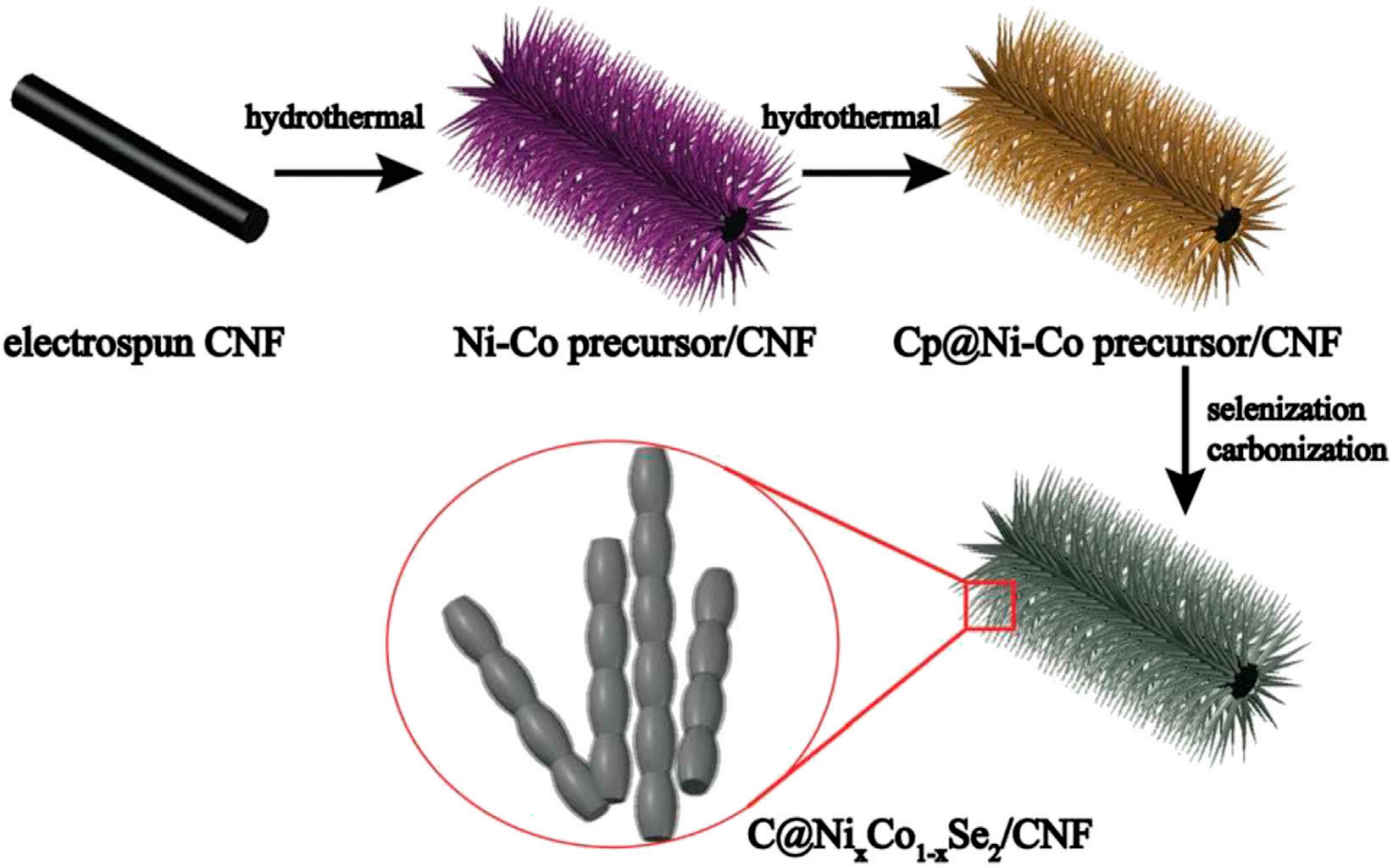
Schematic illustration of the preparation of C coated bimetallic selenides anchored on CNFs.

Wang et al. (2016) first selenized Ni(OH)_2_ nanosheets covered on carbon fiber materials by thermal spray splenization in an argon atmosphere in a quartz tube, and obtained nanostructured electrodes with electrical properties far superior to ordinary carbon fiber materials. Subsequently, the relationship between the reaction temperature and the size of the nanoparticles obtained by the reaction was studied, and it was concluded that in this heat treatment method, as the temperature increased, the size of the nanoparticles obtained by the reaction gradually declined. Wu et al. (2017) first synthesized a carbon fiber precursor coated with Ni-Co nanostructures by the traditional hydrothermal method at 120°C for 12 h (Figure 7). Subsequently, the compound and selenium powder were put into the two ends of the crucible and put into a vacuum quartz tube for heat treatment at 550 °C.

**FIGURE 8:**
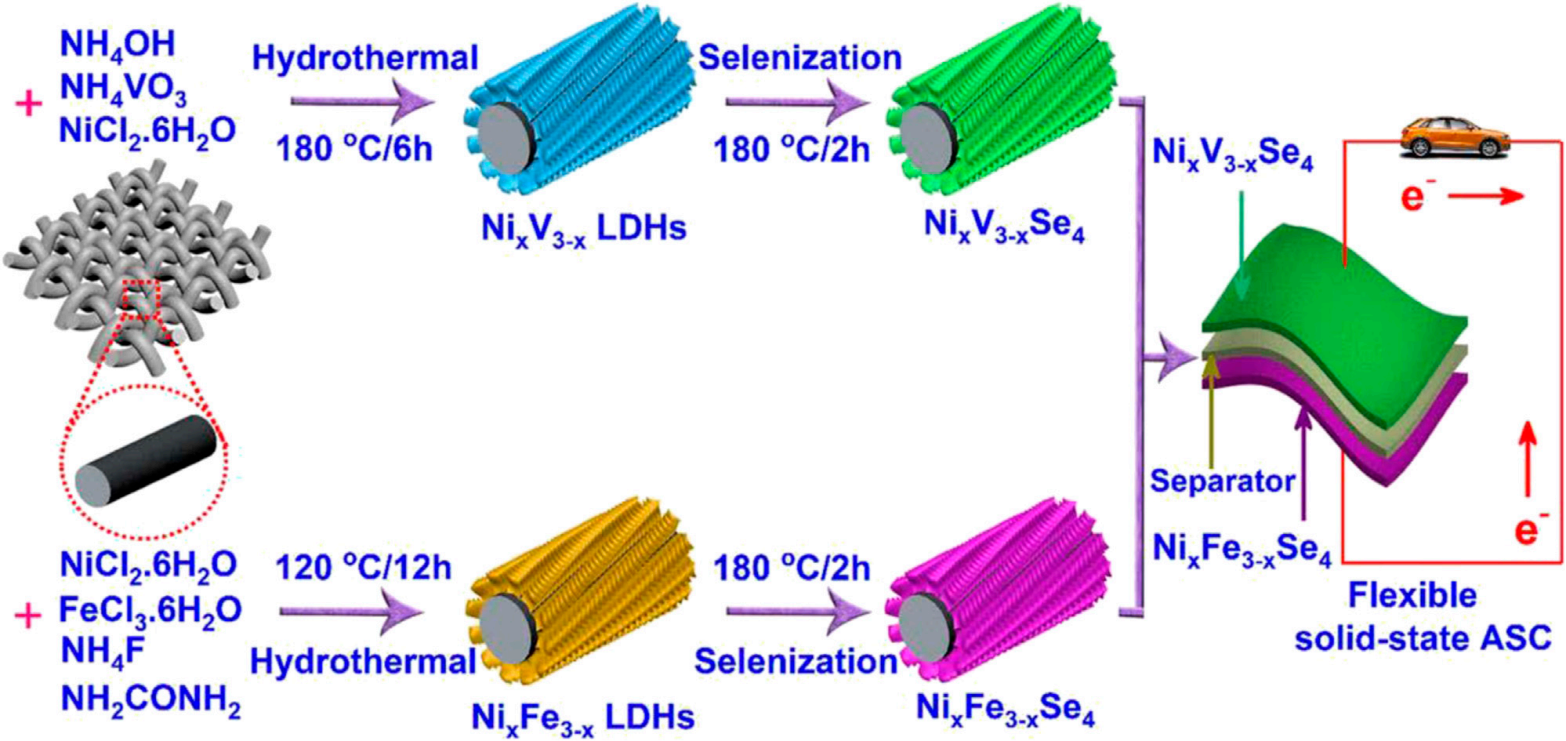
Schematic representation for the design and fabrication of hierarchical Ni_x_V_3−x_Se_4_ and Ni_x_Fe_3−x_Se_4_ nanostructures for solid-state ASCs.

Nguyen et al. (2019) successively synthesized Ni_x_V_3−x_ and Ni_x_Fe_3−x_ layered double hydroxide precursors by traditional hydrothermal method (Figure 8). Then heat treatment is used to perform splenization at 120°C to obtain Ni_x_V_3−x_Se_4_ and Ni_x_Fe_3−x_Se_4_ electrode materials. The flexible SC assembled from these two materials has an energy density of 73.5 Wh/kg at a power density of 0.733 kW/kg, and it still retains 96.6% of the initial capacitance after 10,000 cycles.

## Nickel-Based Selenides Cathode Materials

Since the discovery of the pseudocapacitance energy storage effect, various metal oxides, conductive polymers, and composite electrode materials with carbon materials have always been a focus for SC researchers. In recent years, nickel-based selenide has gradually entered the attention of researchers as the electrode material of SC. Selenium, a transition metal element of the same main group as sulfur and oxygen, has similar chemical properties to that of sulfur and oxygen. Moreover, it has stronger metal properties than sulfur and oxygen. As a result, nickel-based selenide has a higher theoretical specific capacity. Moreover, it also has the characteristics of low cost, rich crust content, and convenient manufacturing, so it has received continuous attention in recent years. It has received continuous attention in the past 2 years. In addition, the slight difference in electronegativity between the two, nickel and selenium can form a wide variety of nickel selenide compounds. Hence, the composition and morphology are the keys to designing new nickel-based selenide nanomaterials.

Nanostructures can provide a direct path and high specific surface area for electron transfer, which will promote the penetration of electrolyte and maximize the utilization of active materials. As is known to all, the chemical properties of electrode materials are determined by the electrons in the outermost layer of atoms. Compared with oxygen or sulfur, selenium has stronger metallicity, larger ion radius, and smaller ionization energy. Therefore, transition metal selenide has better conductivity. Consequently, artificially designed Nano-structured nickel-based selenide becomes a better choice for SC electrode materials.

### Nickel Selenide

To obtain high-performance pseudocapacitance electrode materials, one of the current research directions is to explore the application of transition metal selenide in SC. Although selenium, sulfur, and oxygen are in the same main group, the conductivity of transition metal selenide exceeds that of transition metal oxide and sulfide. The conductivity of sulfide and sulfide. Consequently, SC based on transition metal selenide presents high specific capacitance and outstanding electrochemical performance. As a member of transition metal selenide, nickel selenide can be used in many fields, such as electrochemical catalysis, solar cells, etc. Nickel selenide has good conductivity, a variety of oxidation states, and high theoretical specific capacitance, which makes it a promising candidate for pseudocapacitance electrode materials. In the process of exploring nickel selenide as SC electrode materials, there are problems such as difficult synthetic methods and complicated preparation steps. In recent years, through continuous exploration by scientific researchers, major breakthroughs have been made in solving these issues.

**FIGURE 9:**
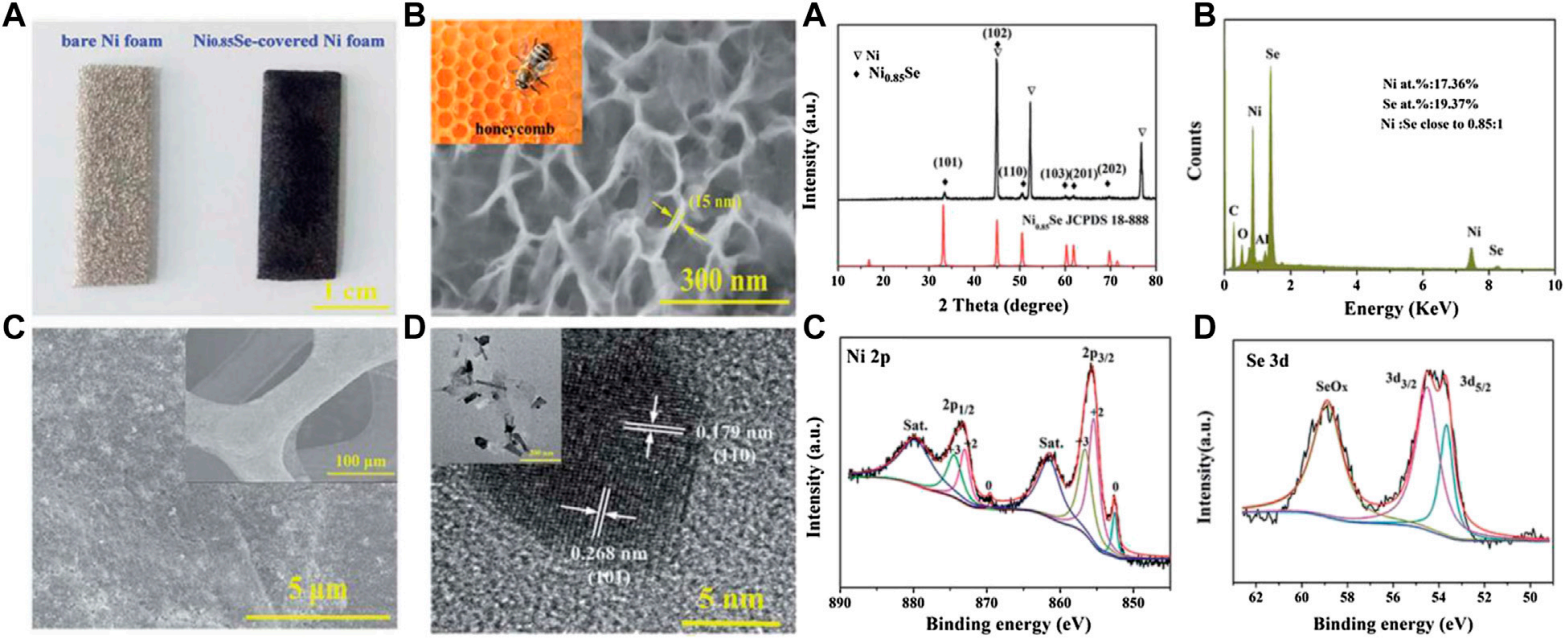
Honeycomb-like metallic nickel selenide nanosheet arrays as binder-free electrodes for high-performance hybrid asymmetric supercapacitors.

Du et al. (2017) used a simple one-step hydrothermal method to grow a honeycomb-like ultra-thin NiSe nanosheet structure as an electrode material (Figure 9) on the surface of a foamed nickel substrate, with the highest specific capacitance at a current density of 1 A/g Reached 3105 F/g, while the specific capacitance is still 1460 F/g at 10 A/g^−1^.

**FIGURE 10:**
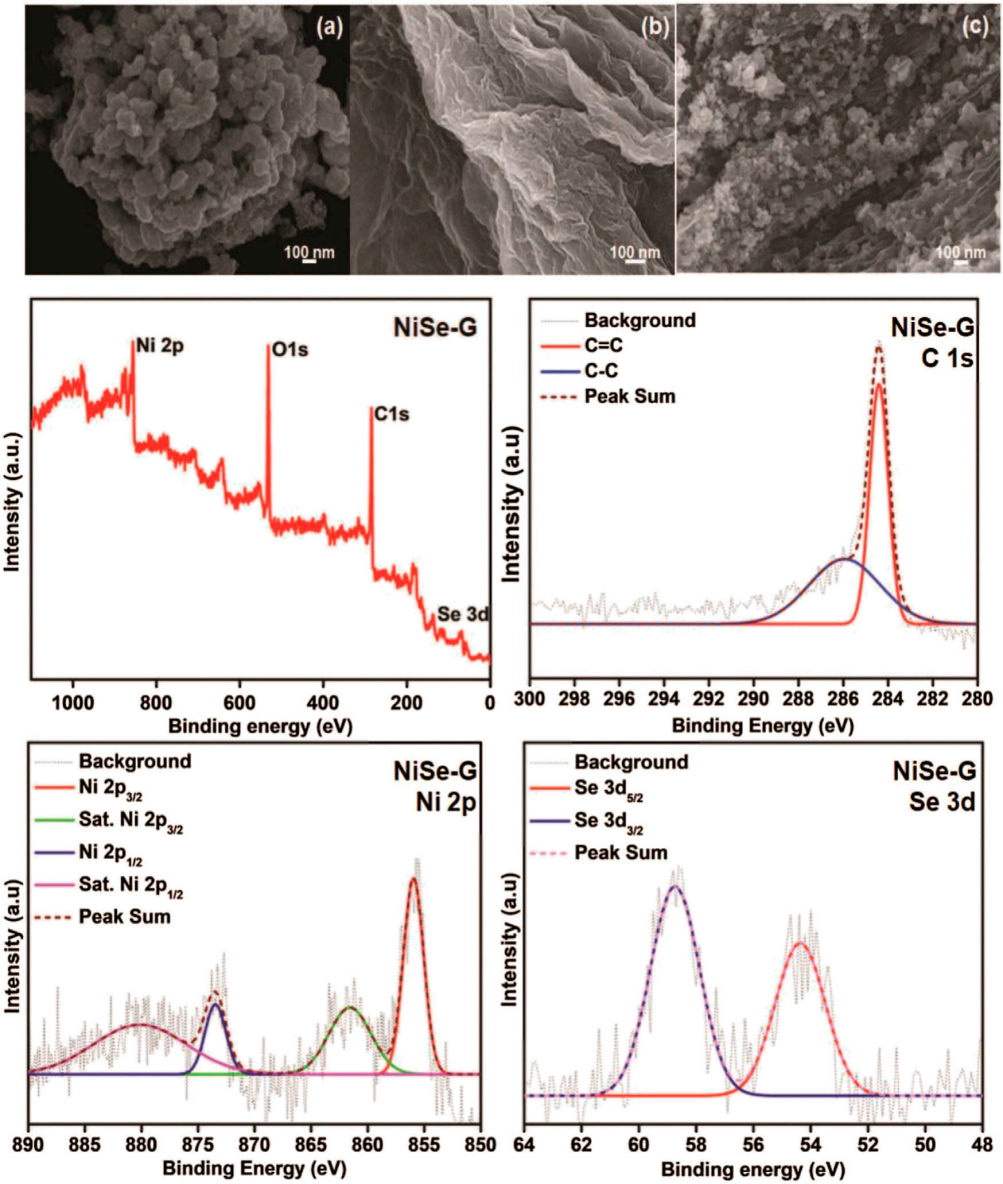
NiSe nanoparticle material with excellent consistency.

Kirubasankar et al. (2018) obtained the NiSe nanoparticle material with excellent consistency on the graphene nanosheets by the *in-situ* hydrothermal method (Figure 10). The electrode has a maximum specific capacitance of 1280 F/g at a current density of 1 A/g. After 2,500 cycles, the attenuation is only 2% compared to the initial value.

**FIGURE 11:**
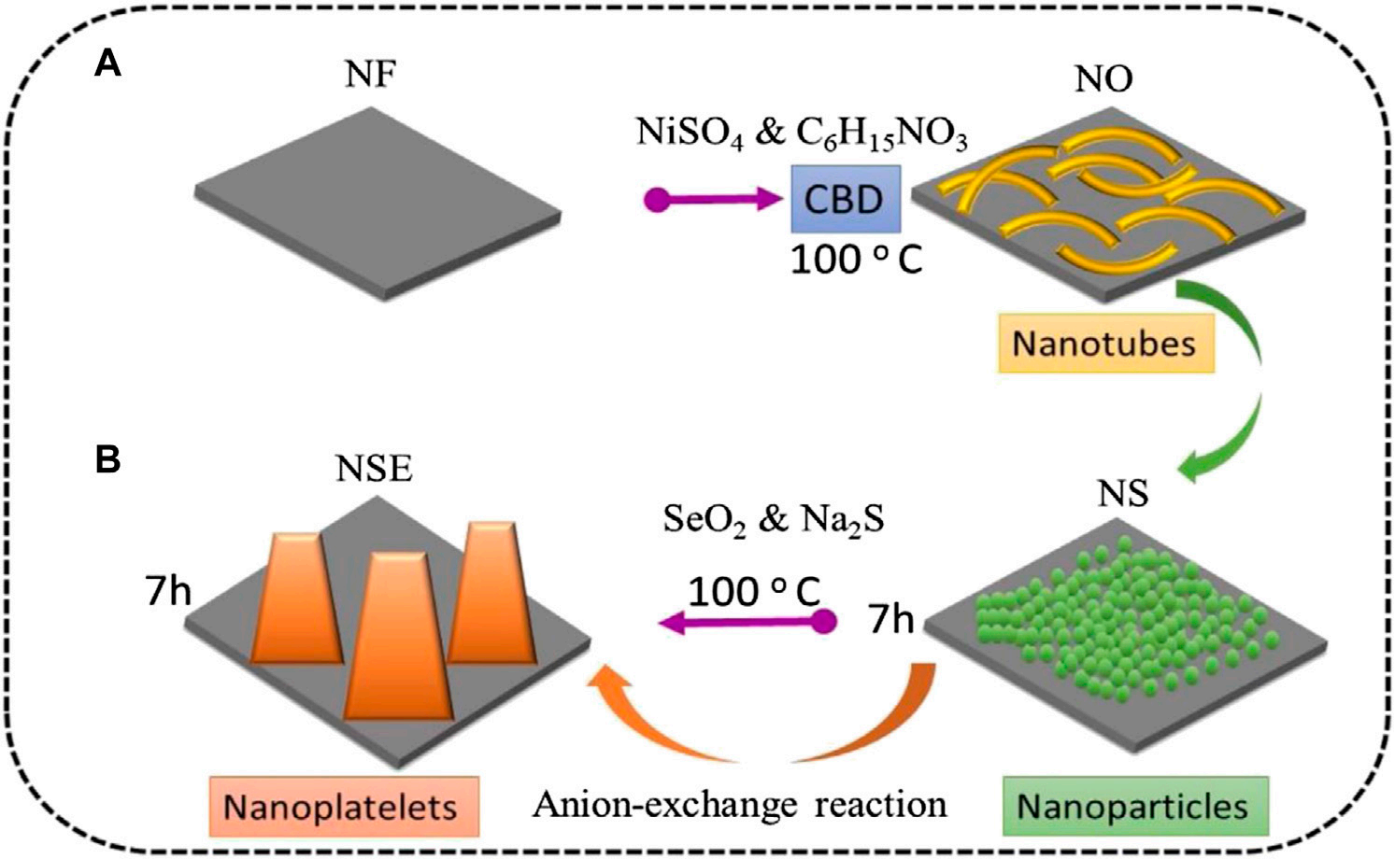
Schematics of **(A)** the self-templating method and **(B)** anion-exchange reaction method.

Mei et al. (2020) designed a self-decomposing nickel-based metal-organic framework layer and prepared a carbon-modified nickel selenide nanocomposite. At 2 A/g, the specific capacitance of this material reached 730 C/g. Thulasi-Varma (Thulasi-Varma et al., 2020) reported the use of chemical bath deposition to vertically grow chalcogenide nickel compounds (oxygen, sulfur, selenium) above the foamed nickel skeleton layer (Figure 11). After comparing the electrical properties of the compounds, it could be determined that the nickel selenide nanomaterials have far better electrochemical properties than nickel sulfide and nickel oxide, and have a capacitance of 2,234.84 F/g at a current density of 10 mA/cm^2^.

Through continuous experimental investigations, researchers have so far developed many nickel selenide cathode materials with excellent performance. Among the cathode materials that have been reported, selenide nanocomposites have the advantages of suitable voltage window, higher theoretical capacity, and stable cycle performance, which capture the attention of the researchers. Nonetheless, there are also some defects in the material. For instance, as the cycle repeats for multiple times, the conductivity of the nickel selenide material would gradually deteriorate and the large volume change might result in the agglomeration of original nanostructure. In response to this situation, a research hotspot has turned to how to reasonably add other components to relieve volume expansion; how to further optimize the nanostructure; how to enhance the electron transmission rate, and ensure good electrochemical performance of the material in the form of nanocomposite materials.

### Nickel-Cobalt-Based Bimetallic Chalcogenides

As a new type of energy storage material, nickel-cobalt-based bimetallic chalcogenides have been extensively studied because of their lower electronegativity, higher electrical conductivity, and electrochemical activity. At present, many scholars have used nickel-cobalt-based chalcogenides as electrode materials in SCs, lithium-ion batteries, dye-sensitized solar cells, and other fields. As an element of the same main group as O and S, Se not only has similar chemical properties as O and S but also has relatively stronger metallicity. Better electronic characteristics will help its application in the field of energy storage. Consequently, in recent years, the application of metal selenide in SC electrode materials has gradually captured more attention. With a rich oxidation state, Selenium, a transition metal element, has better conductivity than S and O (Wang et al., 2013). Besides, because the increase in electrode material components can relieve the cycle fatigue of nickel selenide to a certain extent, nickel-cobalt selenide has also received considerable attention as electrode materials in recent years.

Yang et al. (2019) prepared a nickel-cobalt-selenium-carbon layered nanostructured material with high pseudocapacitance by constructing a binary metal-organic framework, and applied it to a lithium-ion battery. After 30 cycles, it still has 2061 mA h/g capacity.

**FIGURE 12:**
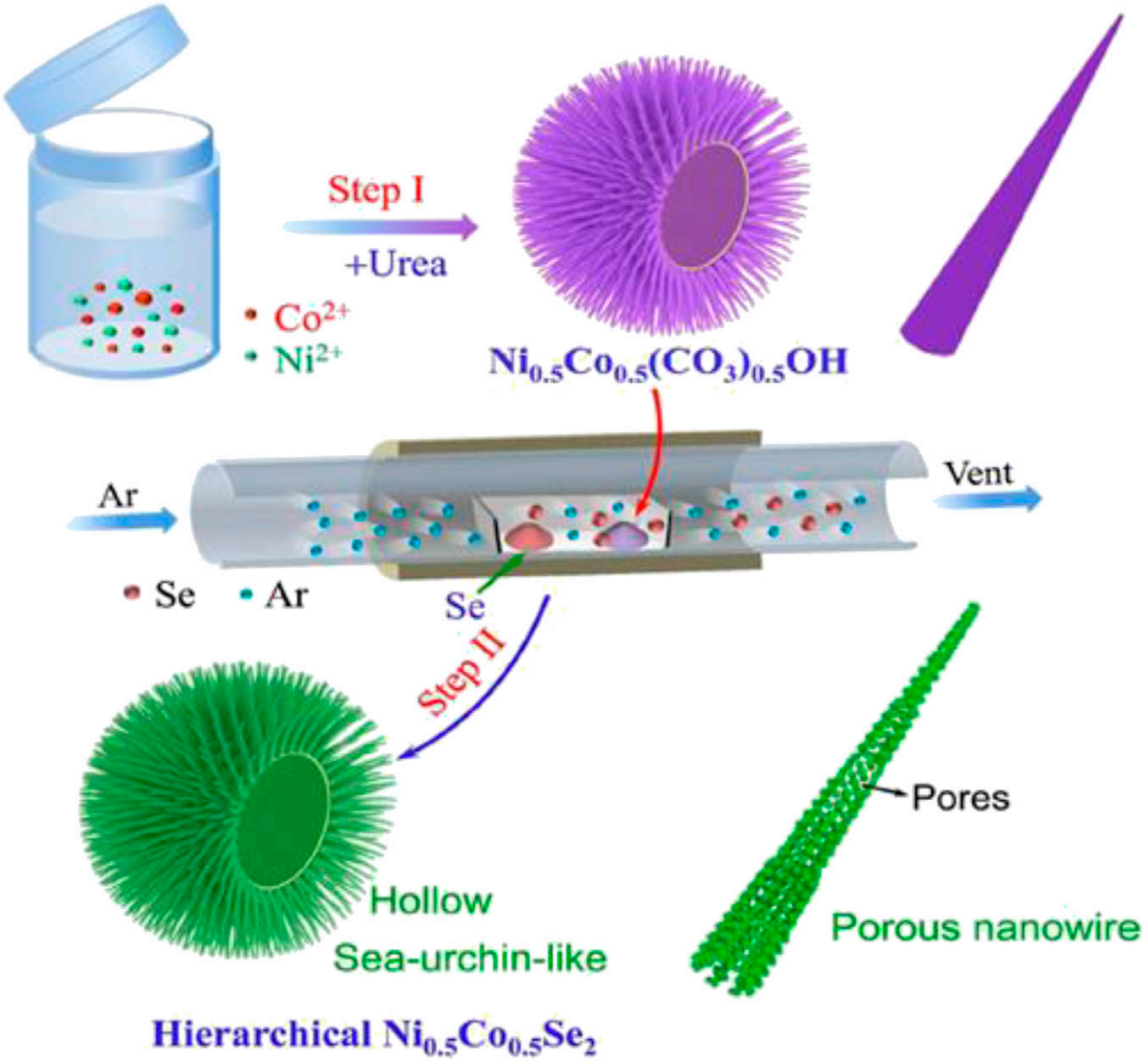
Hierarchical hollow, sea-urchin-like and porous Ni_0.5_Co_0.5_Se_2_ as advanced battery.

Song et al. (2018) synthesized a nanowire material (Figure 12) with a sea urchin-like three-dimensional structure through a low-temperature splenization method, in which the composition ratio of nickel, cobalt, and selenium is Ni_0.5_Co_0.5_Se_2_. The electrode prepared in this way has a capacitance of 524 C/g at 1 A/g, and still retains 91% of the initial capacity after 3,500 cycles. The SC assembled with this electrode material and reduced graphene oxide has a maximum energy density of 37.5 Wh/kg, a maximum power density of 22.2 kW/kg, and cycle stability of more than 10,000 times.

**FIGURE 13:**
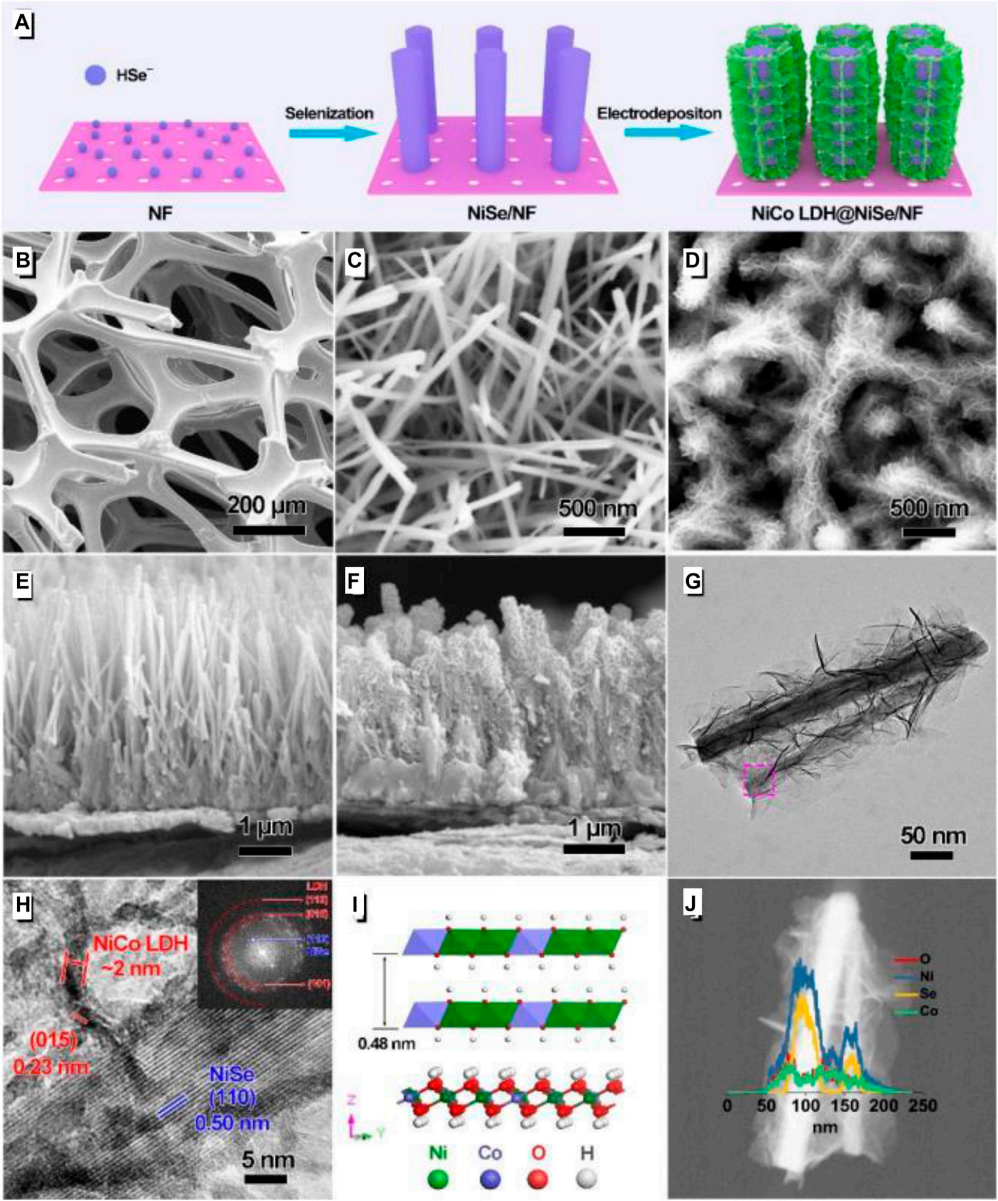
2 nm Thick NiCo LDH@ Nize Single-Crystal Nanorods Grown on Ni Foam as Integrated Electrode.

Lu et al. (2020) designed a layered structure with NiSe single-crystal nanorods as the core, and the outer layer uses nickel foam as the base to grow 2 nm ultra-thin thickness NiCo nanosheets to wrap the core(Figure 13). After testing, the specific capacitance of this electrode material reached 1,131 µAh/cm^2^ at 5 mA/cm^2^. The maximum energy density of the SC assembled with activated carbon reached 0.454 mWh/cm^2^, with the highest power density of 80 mW/cm^2^.

**FIGURE 14:**
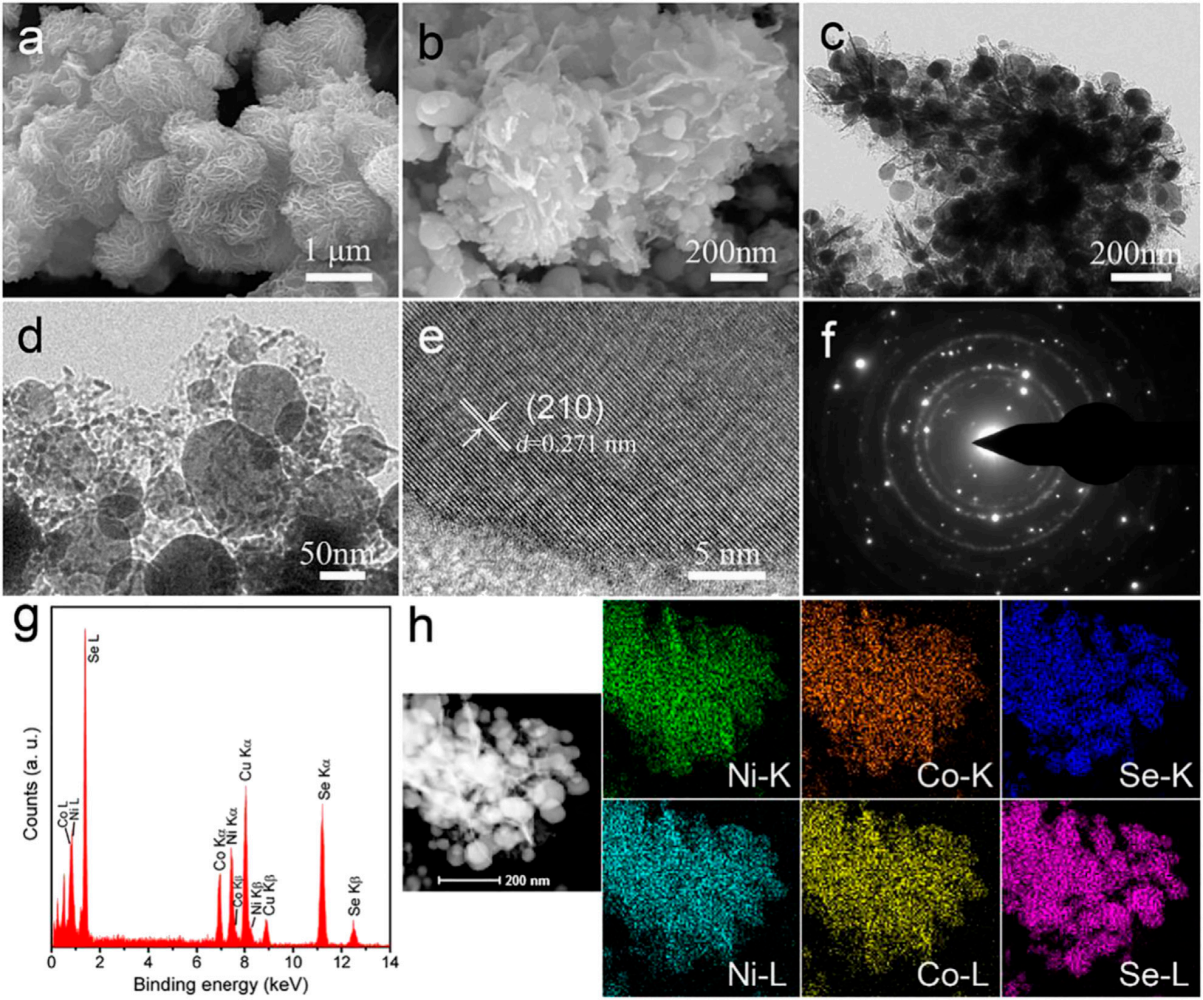
Organometallic framework compound ZIF-7 as the framework.

Qu et al. (2020) used organometallic framework compound ZIF-7 as the framework layer to prepare NiSe_2_/CoSe_2_ bimetallic selenide with a hollow polyhedron structure. This electrode material has a capacitance of 1668 F/g at 1 A/g; the assembled all-solid SC has an energy density of 38.5 Wh/kg under the condition of 802.1 W/kg power density (Figure 14).

### Nickel-M Based Selenide

Nickel-based selenide can be synthesized with many other elements in addition to the binary metal selenide synthesized with the cobalt component as an electrode material. In recent years, this application also receives extensive attention.

Chen et al. (2017) used a gentle single-step hydrothermal method to prepare (Ni,Cu)Se_2_ nanowire materials with extremely high specific surface area and excellent ion mobility through rational design of the structure and control of the material growth time. The asymmetric SC, which is assembled with this material as the positive electrode and reduced graphene oxide material as the negative electrode, exhibited an ultra-high energy density of 44.46 Wh/kg under the condition of a power density of 797.9 W/kg. After 4,000 cycles, the SC still has 97.56% of the initial value.

**FIGURE 15:**
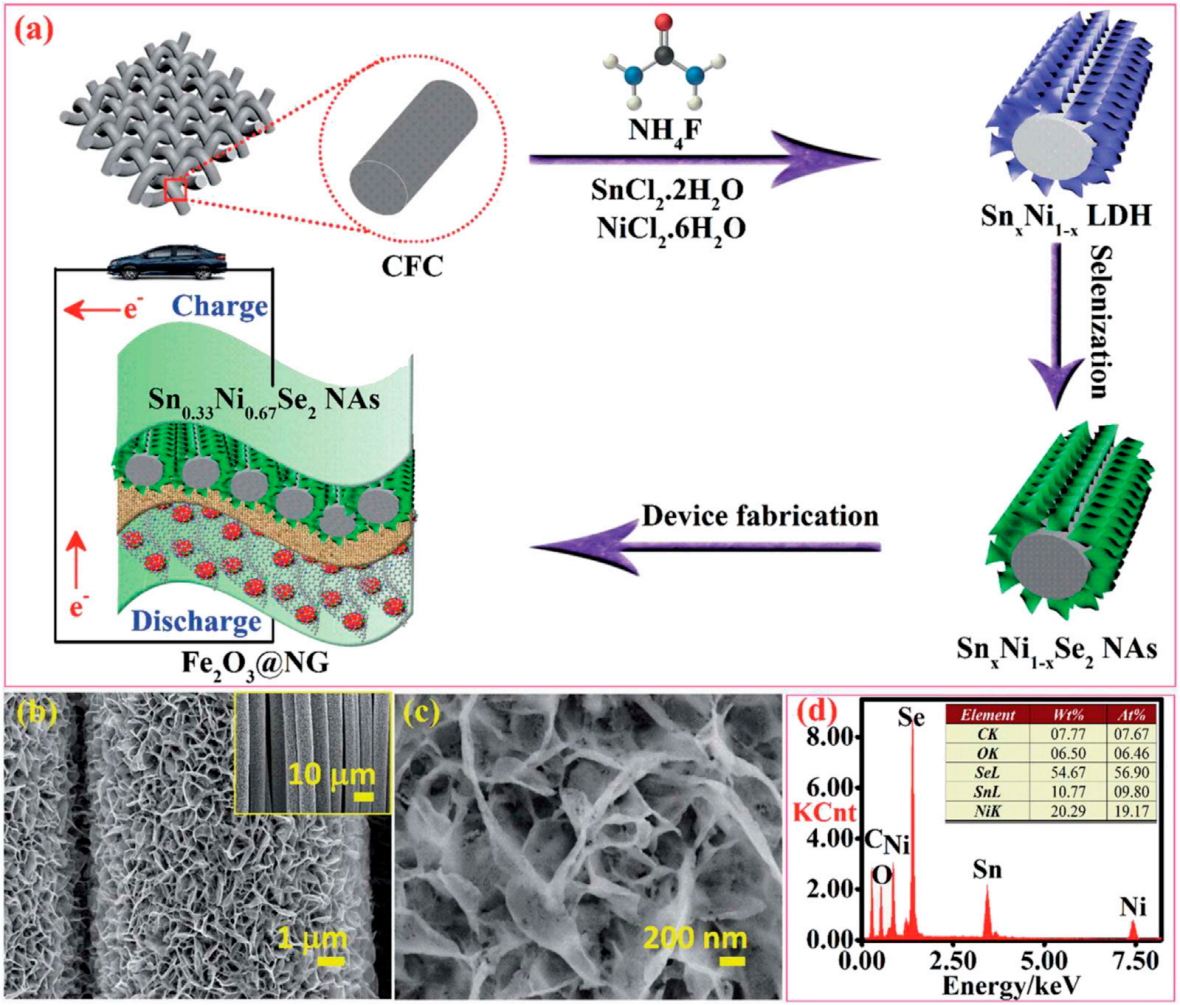
Nickel-tin selenide (Sn_x_Ni_1-x_Se_2_; 0 < *x* < 1) on carbon fiber.

DeepaLakshmi et al. (2019) of Jeonbuk National University synthesized nickel-tin selenide (Sn_x_Ni_1-x_Se_2_; 0 < *x* < 1) on carbon fiber cloth by a simple and cost-effective two-step hydrothermal method. The material has a capacity of 346 mAh/g at a current density of 1.0 mA/cm^2^. The assembled all-solid asymmetric flexible SC has both extremely high energy density and power density (Figure 15).

**FIGURE 16:**
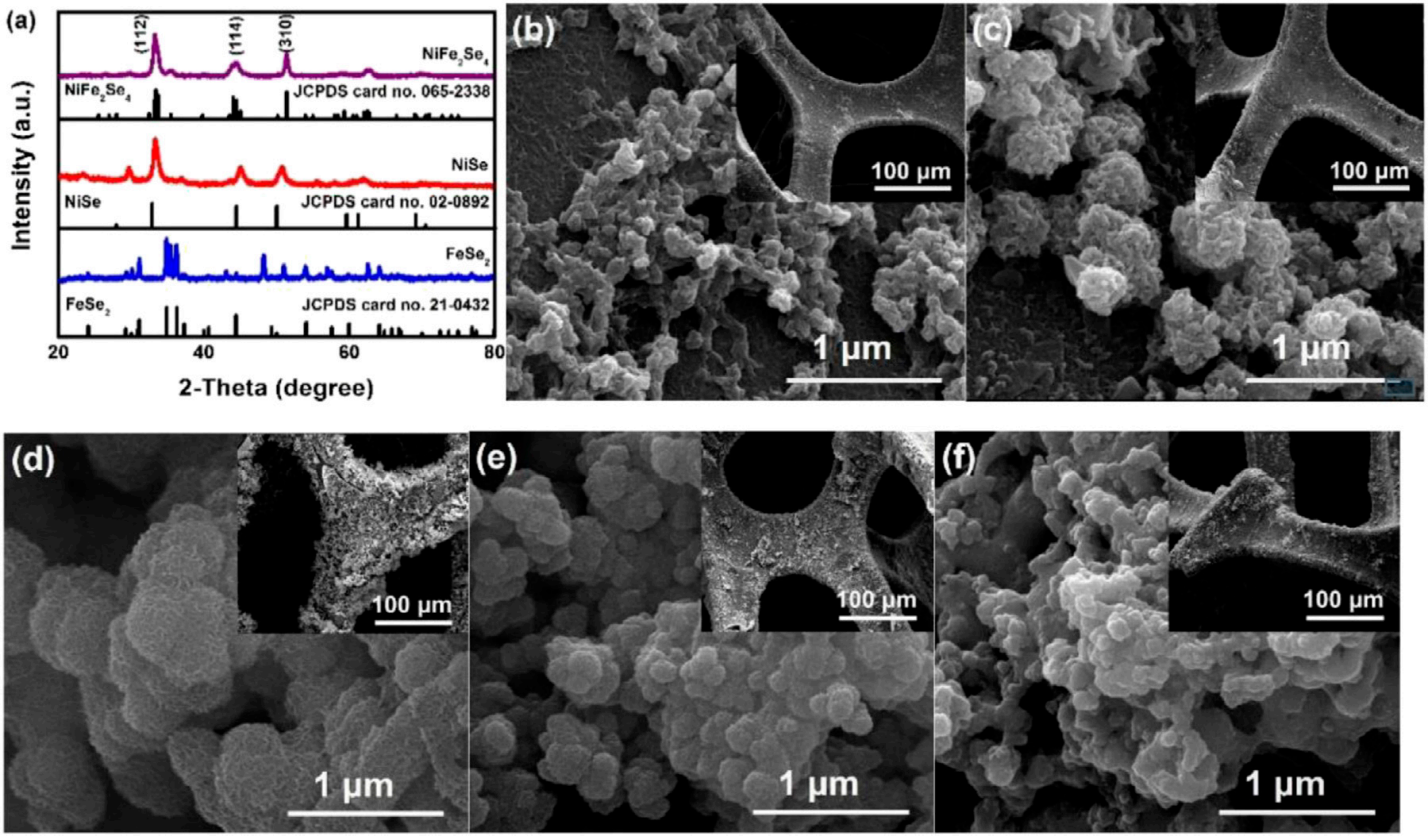
Electrodeposited NiFe_2_Se_4_ on nickel foam as a binder-free electrode for high-performance asymmetric supercapacitors.

Ye et al. (2020a) grew NiFe_2_Se_4_ bimetallic selenide nanoparticle materials with excellent electrical properties on nickel foam by electrochemical deposition (Figure 16). This electrode material not only has the characteristics of good conductivity, low cost, and environmental friendliness, but also benefits from its high roughness and large specific surface area. This material also has a high-speed electron migration rate. The SC assembled with activated carbon reached an energy density of 45.6 Wh/kg at a power density of 800 W/kg. Moreover, the energy density increased to 101.4% of the initial value after 1,000 cycles.

## Material Modification

In materials science, it is a good synthesis path to combine other functional nanomaterials with target materials to obtain composite materials with versatility or improved performance. In recent years, researchers have made some progress in modifying metal chalcogenide nanomaterials with various functional materials (such as carbon materials, noble metals, metal oxides, and other metal selenium compound nanomaterials, etc.). Based on the synergy of these materials, composite materials can generally greatly enhance the performance of the metal selenium compound nanomaterials themselves, which is of practical significance.

Metal chalcogenides usually have similar structures and properties. The modification of metal sulfur selenide mainly exists in the form of metal sulfur selenide-metal chalcogenide heterostructure. For the positive electrode materials of SCs, the existing modification methods mainly include two types: construction of heterostructure modification and carbon substrate modification.

### Heterostructure Modification

For the application of SCs, the electronegativity difference of selenium and nickel is small. Consequently, selenium and nickel can form complexes with different stoichiometry and have different oxidation states, which enables the application of these materials in energy storage systems. Nonetheless, nickel selenide has a short cycle life when used as an electrode. Hence, in recent years, some scholars have focused on the use of nickel selenide and other substances to form composite materials to obtain derivative properties and use them as electrodes in SCs.

Shi et al. (2019) grew a layer of porous flake nickel hydroxide *in situ* on the surface of the nickel selenide nanowire array. The electrode prepared by this material has the excellent conductivity of Ni_3_Se_2_ and the extremely high specific capacitance of Ni(OH)_2_, and the electrode has an electric capacity of 281.5 mAh/g at 3 mA/cm^2^.

**FIGURE 17:**
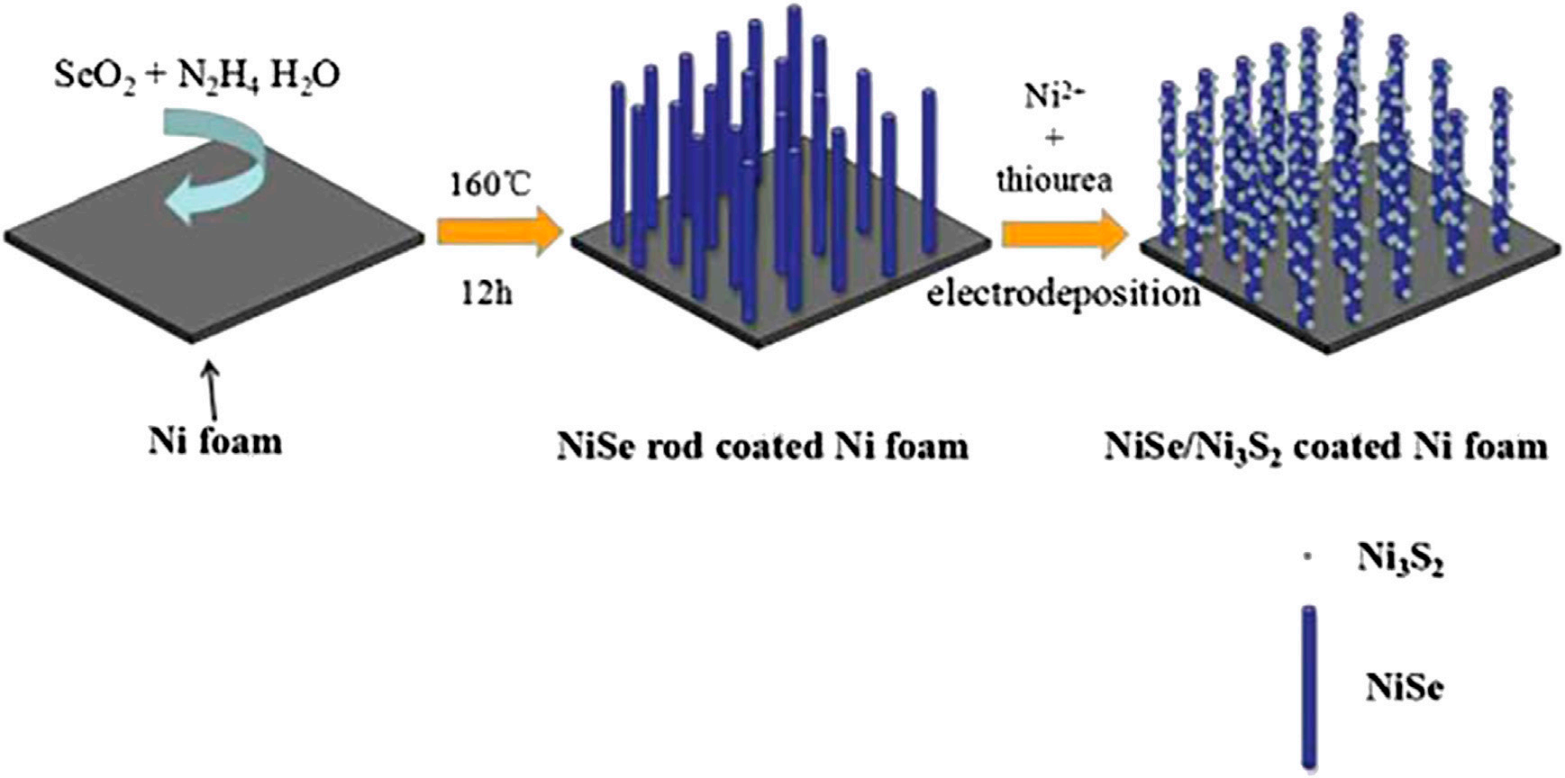
Ni3S2-coated NiSe arrays as positive electrode.

Ye et al. (2019) used a two-step method of splenization and electrochemical deposition to grow a cotton-like nickel sulfide material layer (Figure 17) on nickel selenide nanorods, and obtained higher specific capacitance characteristics than general nickel selenide electrode materials. The SC obtained by assembling this electrode material and activated carbon reaches an energy density of 38.7 Wh/kg at a power density of 192 W/kg and still retains 85.9% of the initial value after 10,000 cycles.

Das et al. (2019) prepared a flower-like nickel selenide cathode material by performing a splenization reaction on porous nickel oxide materials. Besides, the WO_3_@PPy compound was obtained as a negative electrode material by the *in-situ* oxidation reaction of WO_3_ nanorods in the presence of pyrrole. Both electrode materials have better electrochemical properties. The all-solid SC assembled based on these two materials has an energy density of 37.3 Wh kg^−1^ under the condition of a power density of 1,249 Wkg^−1^, and only attenuates 9% after 5,000 cycles.

**FIGURE 18:**
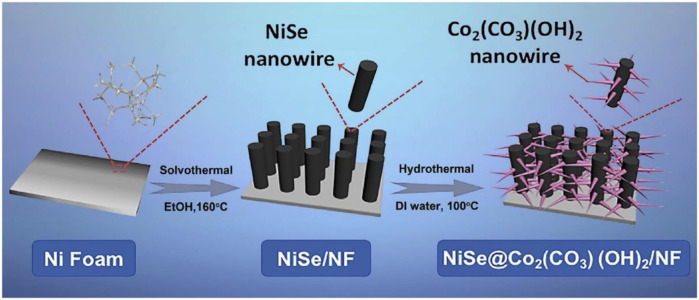
hierarchical NiSe@Co2(CO3)(OH)2 heterogeneous nanowire arrays on Ni foam.

Yuan et al. (2019) first used the hot-melt method to grow the vertical nickel selenide nanowires *in situ* on the nickel foam, and then used the hydrothermal method to grow the crisscrossing Co_2_(CO_3_) (OH)_2_ near the nickel selenide nanowires (Figure 18). The staggering growth of Co_2_(CO_3_)(OH)_2_ nanowires not only avoids the agglomeration of nickel selenide nanowires but also greatly improves the electrical properties of the overall material. The final electrode material NiSe@Co_2_(CO_3_)(OH)_2_/NF has excellent capacitance characteristics. It has a specific capacitance of 9.56 Fem^−2^ at a current density of 4 mA/cm^2^, while still maintaining 68.1% of the initial value when the current density is increased to 80 mA/cm^2^.

**FIGURE 19:**
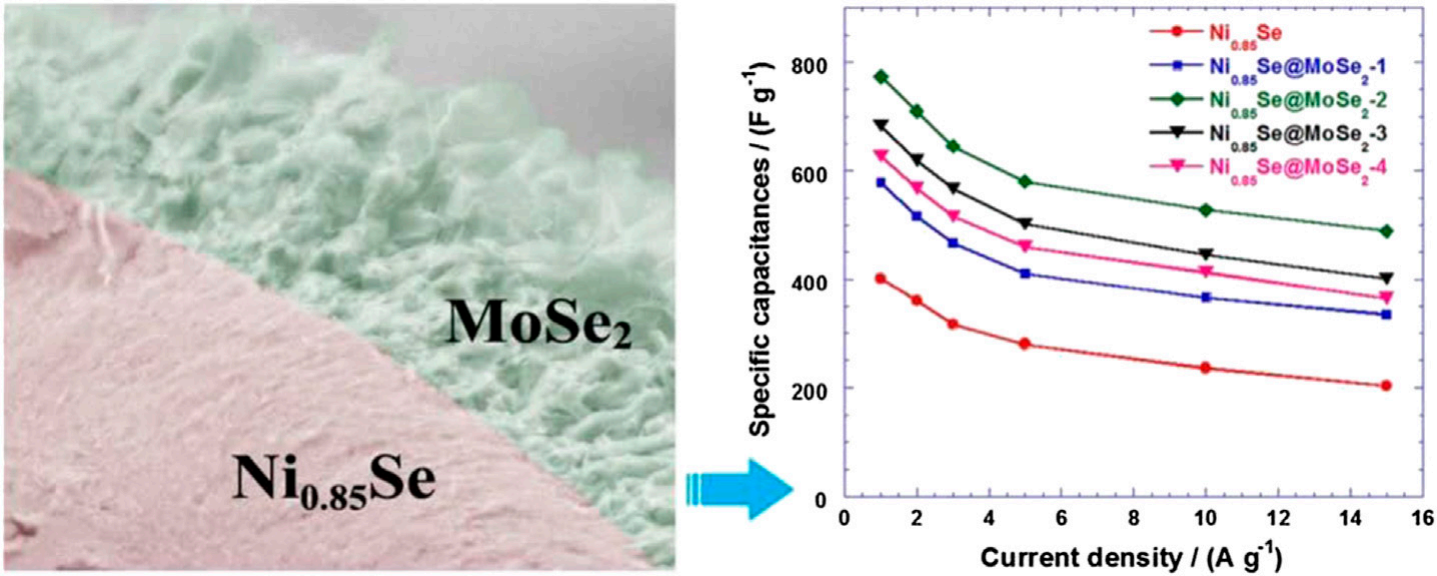
Ni_0.85_Se@MoSe_2_ nanosheet arrays as the electrode for high-performance supercapacitors.

Peng et al. (2017) synthesized Ni_0.85_Se@MoSe_2_ nanosheet array material (Figure 19) on the surface of nickel foam through a gentle one-step hydrothermal method without adding any surfactant. This kind of heterogeneous interface material not only effectively prevents the agglomeration of 2D MoSe_2_ nanosheets, but also acts as an ion buffer layer to protect the electrodes during charge and discharge.

Ye et al. (2020b) prepared NiSe/ZnS hybrid nanostructured materials on the surface of nickel foam by co-electrodeposition. Subsequently, the influence of the ratio of Zn to Ni on the morphology and electrical properties of the material during the preparation process was studied. After determining the optimal ratio, the prepared NiSe/ZnSe layered electrode material has a capacitance of 651.5 mAhg^−1^ at a current density of 1 Ag^−1^. Researchers hold that this excellent electrical performance is mainly owing to NiSe/ZnSe electrodes’ richer redox capabilities, shorter ion migration distances, and more efficient charge transport capabilities than ordinary nickel selenide electrode materials.

### Carbon Substrate

In recent years, carbon materials have attracted great attention from researchers of SC electrode materials for its various advantages such as diversified structures and forms (carbon itself is an allotrope material suitable for electrochemical energy storage), controllable dimensions (its dimensions can be extended from 0 to 3D), low cost, and stable chemical properties (it is stable in acidic and alkaline solutions), a wide temperature range of use, large specific surface area, controllable pore structure and pore size, and environmental friendliness. Carbon materials of different sizes, different forms or allotropes have been widely used in SCs, and graphene materials are at the center of the public attention. Graphene is an advanced two-dimensional carbon nanomaterial composed of carbon atoms arranged in a hexagonal network. It has many advantages, such as high thermal conductivity, high electron mobility, and large specific surface area. Nevertheless, in the process of preparing graphene, the specific surface area is easily reduced due to stacking. The graphene needs to be further activated so that they are separated, and the specific surface area of the active material is maximized. Annealing at high temperatures can produce graphene oxide separated from each other. The synthesis of reduced graphene oxide/activated carbon composite materials has been achieved in one step in recent years. The specific surface area of this composite material after annealing is almost eight times that of the original graphene oxide. And its electrochemical performance has been significantly improved.

**FIGURE 20:**
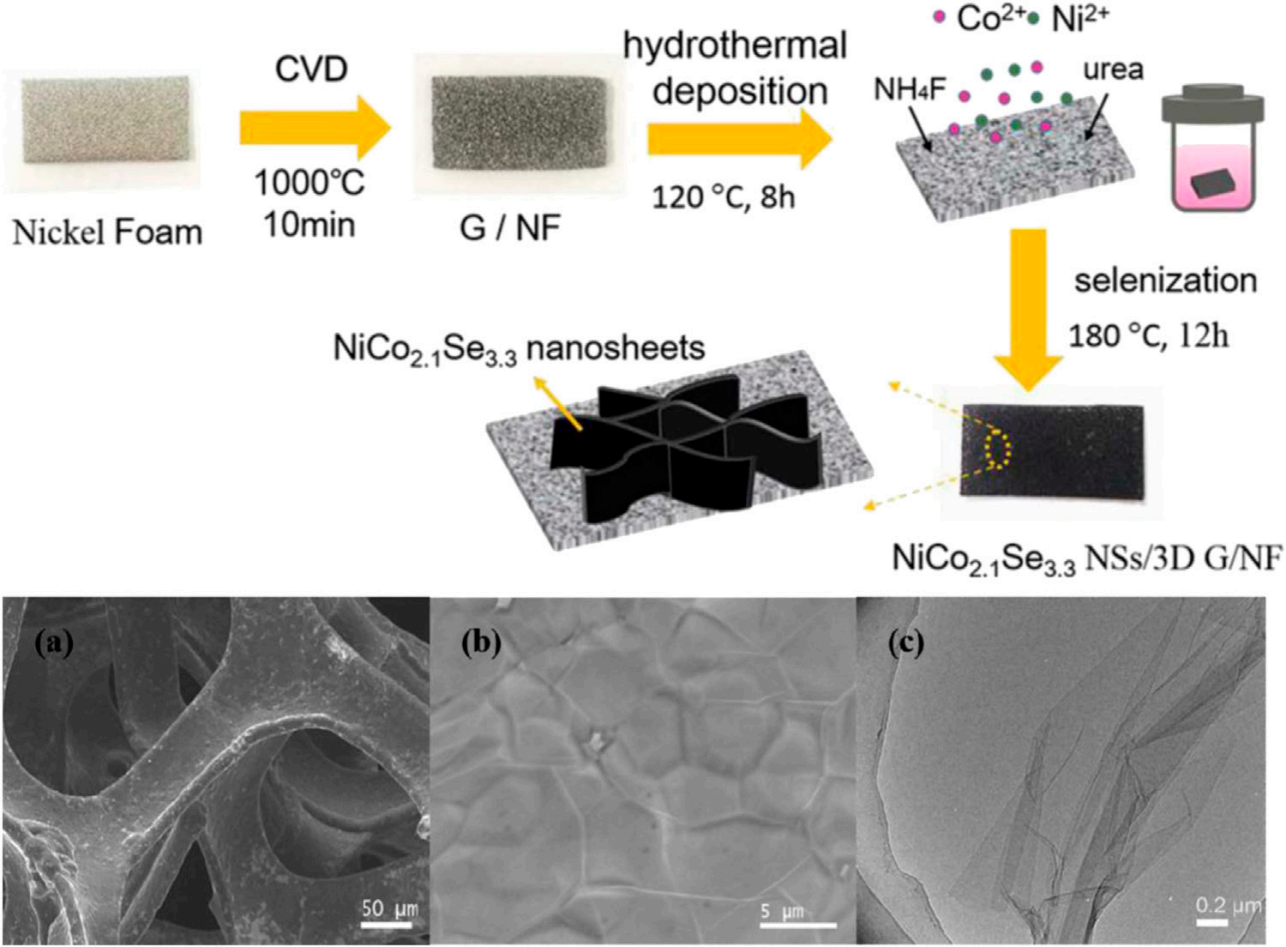
Schematic drawing of the preparation process of NiCo2.1Se3.3 NSs/3D G/NF binder-free electrode.

Wang et al. (2019) uniquely used chemical vapor deposition (CVD) to grow a layer of graphene with a 3D structure using methane as a carbon source. Then, a two-step hydrothermal method was used to grow the Ni-Co bimetallic precursor and complete the splenization reaction (Figure 20). The ratio of Ni-Co elements in the process was optimized after a comparative test. The final NiCo_2.1_Se_3.3_ nanosheets/3D Graphene/nickel foam material achieved an excellent capacitance of 742.4 F/g at a current density of 1 mAcm^−2^. Meanwhile, when the current density is increased to 10 times, it still has a capacitance of 471.78 F/g, and after 10,000 cycles, it still has 83.8% of the initial capacitance.

Gu et al. (2019) combined the hydrothermal method and the hot-melt approach. First, the Ni(OH)_2_ precursor was prepared by the hydrothermal method, and then a mixture of nitrogen-doped reduced graphene oxide and nickel selenide was prepared by the hot-melt method. During the preparation process, the relationship between the dosage of reduced graphene oxide and the performance of the prepared electrode material was discussed. The optimized electrode material has a capacitance of 2,451.4 F/g at a current density of 1 A/g. The SC assembled by activated carbon and this electrode material has an energy storage capacity of 40.5 Wh/kg under the condition of an energy density of 841.5 W/kg.

Chen et al. (2020) prepared a tightly wound NiSe electrode material on the jungle-like carbon nanotube skeleton layer by electrodeposition. The maximum energy storage of the SC assembled with this material and graphene reaches 32 Whk/g, and the highest power density is 823 Wk/g. At the same time, this electrode material has certain application prospects in the electrolysis of water.

Sitaaraman et al. (2020) prepared a series of nickel selenide nanocomposites by hydrothermal method, including ordinary graphene nickel selenide hybrid materials, nitrogen-doped graphene nickel selenide hybrid materials, and boron-doped graphene selenide Nickel mixed material. Subsequently, the three materials were characterized by morphological characterization and voltammetry and other electrical performance tests. It is found that the nitrogen-doped graphene nickel selenide material has the best electrical properties. After discussion, researchers believe that nitrogen-doped graphene has a consistent and synergistic effect.

**FIGURE 21:**
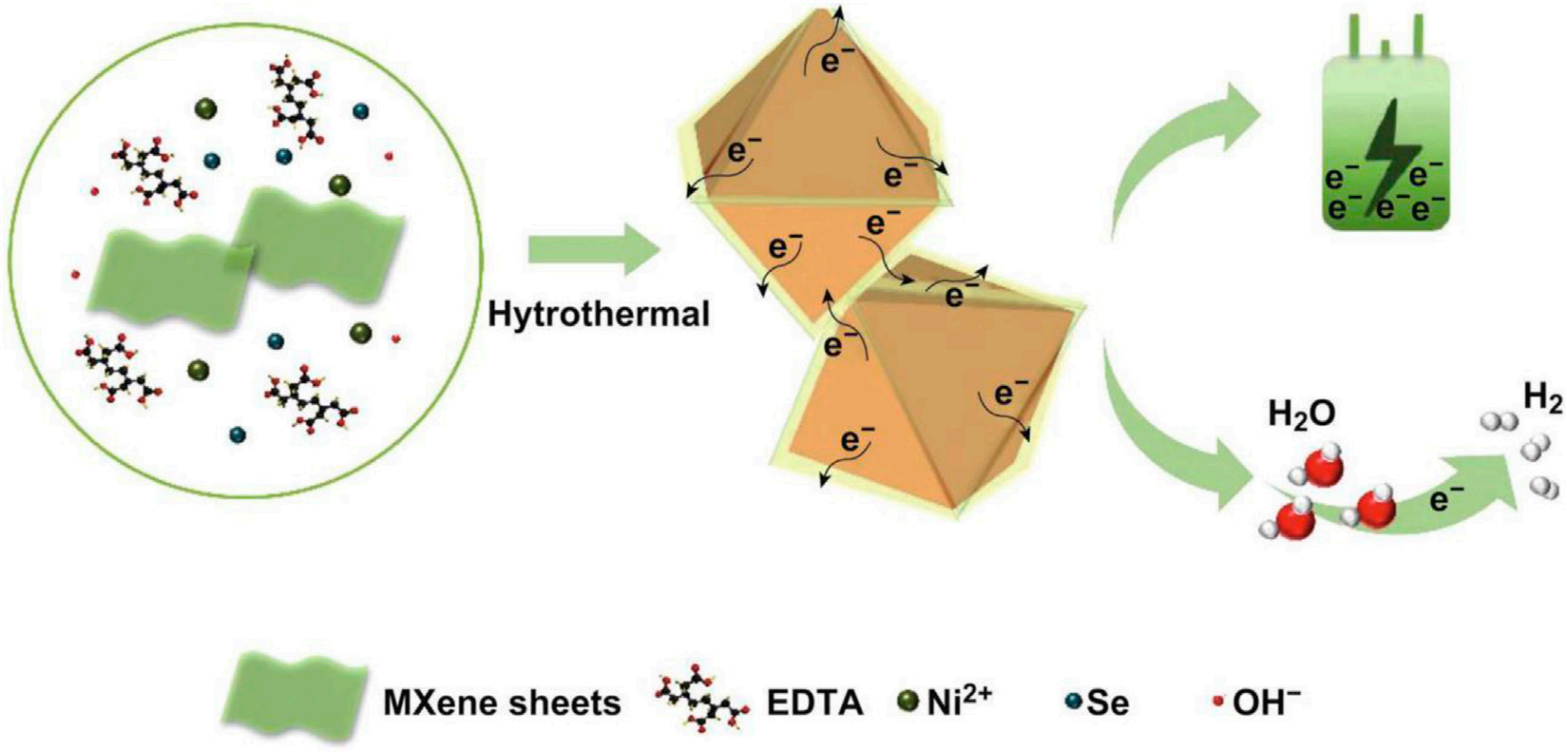
Schematic illustration for the formation of octahedral NiSe2/Ti3C2Tx hybrid through one-pot hydrothermal method.

In addition to participating in the synthesis of SC electrodes in the form of graphene, two-dimensional transition metal carbonitrides (MXene) is also an application method of carbon materials in SC electrodes. MXene has the advantages of good hydrophilicity, high chemical stability, adjustable interlayer spacing, and high electronic conductivity, which renders it a broader application prospects in the field of SCs. In recent work, Jiang's (Jiang et al., 2019) research group innovatively introduced MXene nanomaterials. By wrapping ultra-thin Ti_3_C_2_T_x_ MXene nanosheets on the outer layer of nickel selenide with an octahedral crystal structure, an electrode material with outstanding electrical properties and ultra-high cycle stability is prepared, which can be used not only in SCs but also in hydrogen evolution reactions (Figure 21).

### Doping

In the field of electrode materials, the surface coating is usually one of the effective strategies to improve the electrochemical performance of layered cathode materials (Chen et al., 2018; Li et al., 2018; Zhao et al., 2019; Li et al., 2020; Liang et al., 2020). Nonetheless, a single surface coating is difficult to fundamentally stabilize the crystal structure of the material. Experiments and theoretical calculations show that heteroatom doping can significantly improve the stability of the crystal structure of the material (Kong et al., 2019; Wu et al., 2019; Yu et al., 2019; Zhu et al., 2020). Consequently, doping is also a critical modification and performance improvement method for the cathode material of SCs.

**FIGURE 22:**
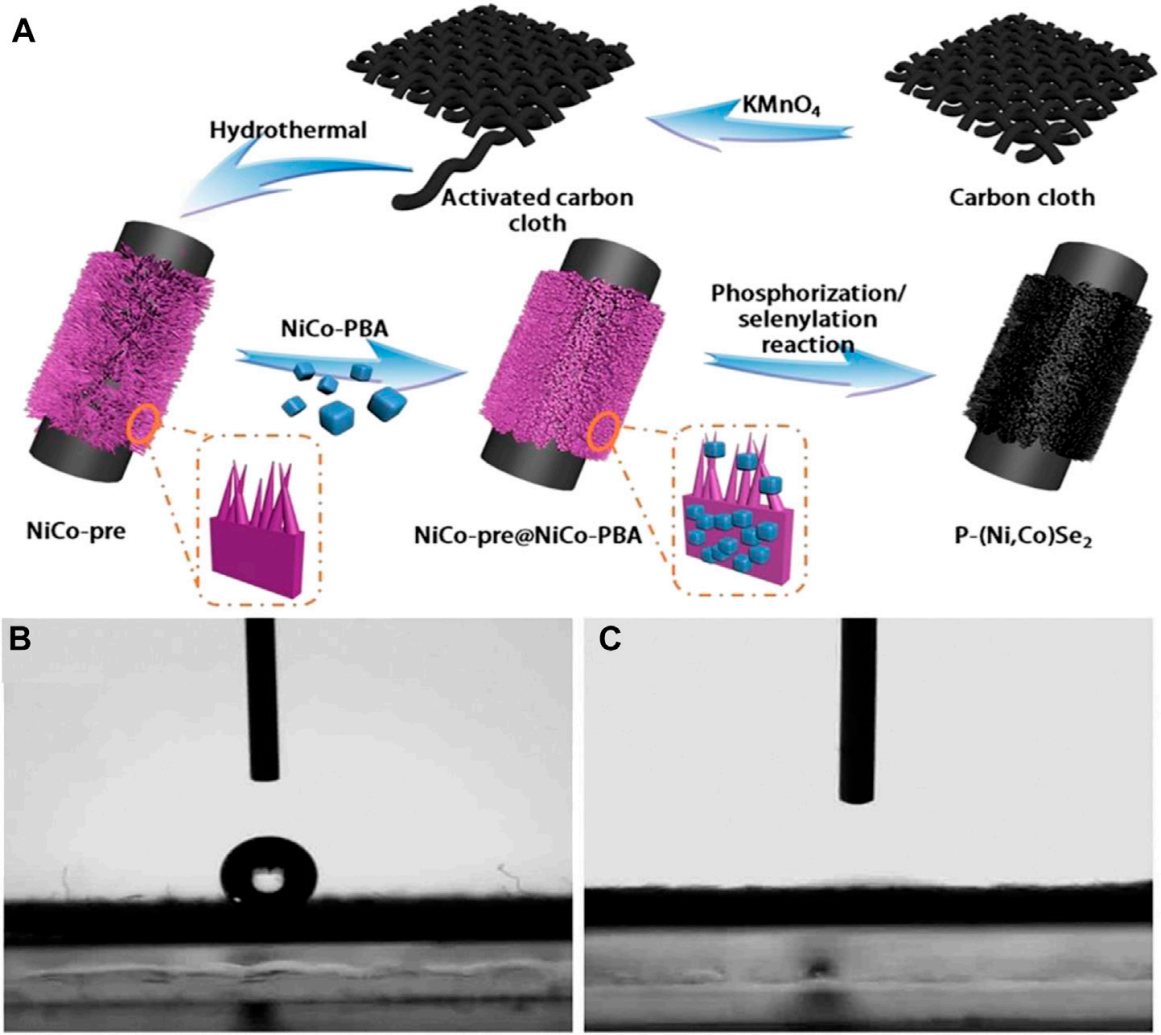
Schematic illustration of the fabrication procedure of P-(Ni,Co)Se_2_ NAs on activated carbon cloth.

Zong et al. (2020), designed an electrode material with a special structure. First, the researchers used potassium permanganate to activate the activated carbon fiber cloth. Afterward, a gentle single-step hydrothermal method was used to grow NiCo precursor nanoarrays (nanoarrays, NAs including nanosheets and nanoflakes) on the surface of the carbon fiber cloth. Subsequently, Prussian blue derivative nanoparticles were grown on the surface of the precursor by the liquid phase method, and its main function was to provide anchor points for the subsequent phosphide splenization reaction. Finally, the phosphide splenization reaction of the precursor was completed by the thermal decomposition method in the quartz tube to obtain P-(Ni, Co)Se_2_ NAs electrode material (Figure 22). Researchers have characterized and analyzed the prepared materials, which leads them to the conclusion that there are advantages as follows:

(1) The uniform distribution of the hollow Prussian blue derivative nanoparticles and the neat nanoarray structure in the material not only greatly increases the surface area of the material, but also makes the material have stronger nanostructure stability at the same time.
(2) There is no need for adhesion during the material synthesis process, which greatly reduces the charging resistance of the material itself and improves the conductivity.
(3) Doping P element into nickel-cobalt selenide renders the material more electrically active sites and improves the electron transfer efficiency.

**FIGURE 23:**
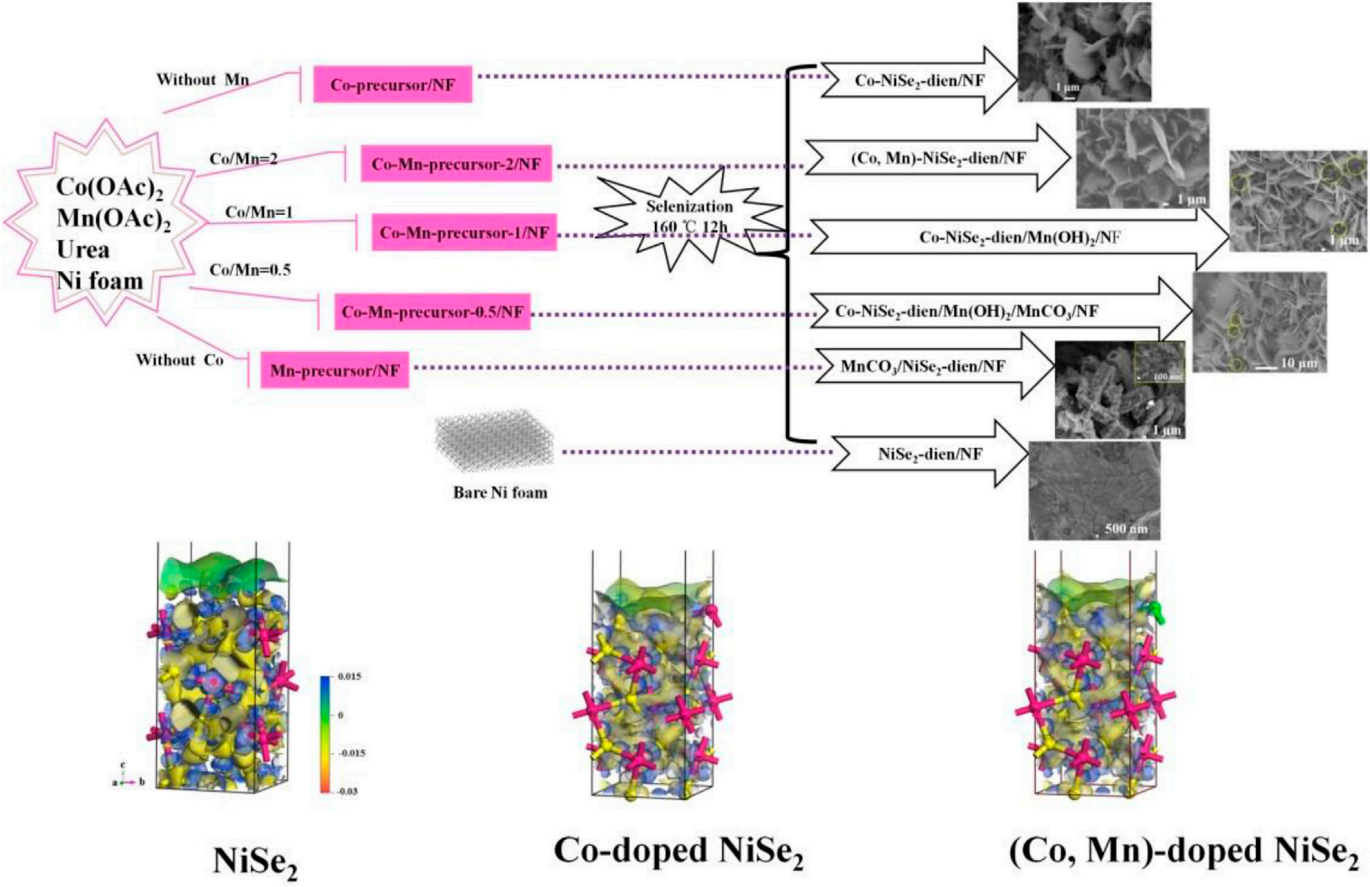
(Co, Mn)-doped NiSe_2_-diethylenetriamine (dien) nanosheets and (Co, Mn, Sn)-doped NiSe2 nanowires for high performance supercapacitor.

The prepared P-(Ni, Co)Se_2_ NAs electrode material has a capacitance of 755 C/g under the condition of 2 mAcm^−2^. The hybrid SC, which is assembled with P-(Ni, Co)Se_2_ NAs electrode material and the ZIF-8 derived carbon material, exhibits a capacitance of 45 Wh/kg under the condition of a power density of 446.3 W/kg.

Dan et al. (2019) grew a series of Co-Mn doped nickel foam precursors with different composition ratios through a one-step hydrothermal method. Afterward, the hot-melt method is adopted to carry out the splenization reaction. Different from others, a certain amount of diethylenetriamine (dien) is added during the reaction in this experiment. Discussing the influence of Co and Mn doping on the morphology of nanomaterials, the researchers designed a series of control experiments with different Co-Mn doping ratios according to the composition ratio. From the experimental results, the doping ratio of Co-Mn has a huge impact on the prepared nanostructures. In addition, when Mn atoms are individually doped, a hollow nanotube structure is prepared by many nanowires intertwined, and a sheet-like nanostructure is gradually produced after Co atoms are added as doping components. Consequently, the morphology of the prepared nanomaterials can be controlled by adjusting the doping ratio of Co-Mn. Researchers tend to believe that the Co element promotes the growth of metal selenide nanomaterials toward its 011-crystal plane (thickness direction), thus promoting the generation of sheet-like nanostructures. Among various doping ratios of the control group, the (Co, Mn)-NiSe_2_-dien/NF prepared after Co-Mn doping in a 2:1 composition ratio has the best electrical properties. This material has a specific capacitance of 288.6 mAh/g under the condition of 1 A/g Subsequently, after deliberation, the researchers hold that the material doped with this component not only has an ultra-flaky nanostructure, but the addition of Co-Mn and dien makes the current density center position around the Ni and Se material redistribute.

## Conclusion and Perspectives

The energy issue is an issue that the national society attaches great importance to. The rapid consumption of non-renewable energy has made the development of new and efficient energy storage equipment the focus of attention. As the contradiction between economic development and environmental problems becomes increasingly serious, electrochemical energy storage devices have been the concentration. Scientists have devoted to the experiments and exploration of the advantages of the SC such as high specific capacity, high power density, fast charging speed, good ultra-low temperature characteristics, long cycle life, and environmental friendliness. However, there are still impediments in the wider use of the SC, such as the large leakage current, low monomer energy density, low monomer voltage, low degree of industrialization, and high prices of some electrode materials. Moreover, compared with commercial batteries, the energy density of existing carbon material the SC is still relatively low. In order to solve this low energy density problem, battery-the SC has been extensively studied, which combines Faraday electrodes (as an energy source) and capacitor electrodes (as a power source) to increase the operating voltage. Whether a suitable material can be developed to promote the balance of rapid charge movement between the two electrodes is the key to achieve high energy and power density. Electrode materials have a great impact on the electrochemical performance of SCs. Consequently, many researchers have taken the preparation of new electrode materials and improving the electrochemical performance of electrode materials as the research direction (Tan et al., 2020a; Wang et al., 2020a; Tan et al., 2020b; Wang et al., 2020b; Gan et al., 2020; Zhang et al., 2020). Nickel-based selenide has a unique electronic structure and high specific capacitance and is considered to be the most promising electrode material for the next generation of the SC among many pseudo capacitance electrode materials.

In recent years, many researchers have carried out in-depth research on nickel selenide nanomaterials as electrodes for the SC and have also made great progress. In the future, the SC will receive increasing attention in energy storage. To explore and develop the performance and practical application of its electrode materials in many ways requires more scientific knowledge and efforts. As far as the author is concerned, the research of nickel-based selenide as a SC cathode material has a good research prospect in the following directions in the future:

(1) The characterization of nickel-based selenide and its composite materials could reflect its structure, morphology, and electrochemical performance after the electrochemical reaction. Through comparative analysis, an in-depth study should be implemented to investigate the changes in the structure and performance of nickel-based selenide materials in the electrochemical process. Based on the analyses, an attempt should be made to further optimize the relative performance and microstructure of the electrode material.
(2) Based on the research on carbon-based nickel-based selenide electrode materials, researchers should try to further compound with double-layer capacitor materials (graphene, carbon nanotubes, etc.). The rate performance and cycle stability performance of the nickel selenide electrode can be further improved by utilizing the excellent cycle performance of the electric double layer capacitor material and the synergy between the electric double layer capacitor material and the nickel-based selenide.
(3) In recent years, the application of wearable and foldable electronic products has brought extensive attention to flexible energy storage devices. Therefore, the construction of nickel-based selenide nanoarrays on flexible substrates (such as carbon cloth) will have excellent market prospects. In addition, the flexible substrate can further enhance the electrical properties of the electrode material, because the inherent structural characteristics of the flexible substrate can effectively prevent the agglomeration of nanoparticles.
(4) The mechanism of action between the nickel-based selenide electrode material and the electrolyte system should be further studied to explore the best system between the electrode and the electrolyte to obtain a hybrid SC with better performance.

## Author Contributions

All authors listed have made a substantial, direct, and intellectual contribution to the work and approved it for publication.

## Conflict of Interest

The authors declare that the research was conducted in the absence of any commercial or financial relationships that could be construed as a potential conflict of interest.
